# Key HPI axis receptors facilitate light adaptive behavior in larval zebrafish

**DOI:** 10.1038/s41598-024-57707-6

**Published:** 2024-04-02

**Authors:** Han B. Lee, Soaleha Shams, Viet Ha Dang Thi, Grace E. Boyum, Rodsy Modhurima, Emma M. Hall, Izzabella K. Green, Elizabeth M. Cervantes, Fernando E. Miguez, Karl J. Clark

**Affiliations:** 1 Department of Neurology, Mayo Clinic, Rochester, MN USA; 2 Department of Biochemistry and Molecular Biology, Mayo Clinic, Rochester, MN USA; 3 Department of Agronomy, Iowa State University, Ames, IA USA; 4 Center for Individualized Medicine, Mayo Clinic, Rochester, MN USA; 5 Neuroscience, Mayo Clinic Graduate School of Biomedical Sciences, Mayo Clinic, Rochester, MN USA; 6 Present Address: Department of Animal Science, Texas A&M University, College Station, TX USA

**Keywords:** GR (glucocorticoid receptor), Stress response, Zebrafish, Light assays, Light adaptation, GAM (generalized additive models), Stress and resilience, Endocrinology, Homeostasis

## Abstract

The vertebrate stress response (SR) is mediated by the hypothalamic–pituitary–adrenal (HPA) axis and contributes to generating context appropriate physiological and behavioral changes. Although the HPA axis plays vital roles both in stressful and basal conditions, research has focused on the response under stress. To understand broader roles of the HPA axis in a changing environment, we characterized an adaptive behavior of larval zebrafish during ambient illumination changes. Genetic abrogation of glucocorticoid receptor (*nr3c1)* decreased basal locomotor activity in light and darkness. Some key HPI axis receptors (*mc2r* [ACTH receptor], *nr3c1*), but not *nr3c2* (mineralocorticoid receptor), were required to adapt to light more efficiently but became dispensable when longer illumination was provided. Such light adaptation was more efficient in dimmer light. Our findings show that the HPI axis contributes to the SR, facilitating the phasic response and maintaining an adapted basal state, and that certain adaptations occur without HPI axis activity.

## Introduction

The stress response (SR) is defined as the body’s response to actual or perceived threats. The SR is mediated by the hypothalamic–pituitary–adrenal (HPA) axis and has evolved in vertebrates. When a stimulus reaches the paraventricular nucleus (PVN) of the hypothalamus, the SR is initiated in a hormonal cascade starting with the secretion of corticotropin releasing hormone by the PVN. Subsequently, the anterior pituitary releases adrenocorticotropic hormone (ACTH) that binds its receptor, MC2R (melanocortin receptor type 2) on the adrenal gland. The adrenal gland in turn secretes the effector molecule glucocorticoids (GCs; cortisol for humans and fish; corticosterone for rodents). GCs travel throughout the body and effectuate the SR by binding to their cognate receptors (corticosteroid receptors [CR] that include glucocorticoid receptor [GR; *NR3C1*] and mineralocorticoid receptor [MR; *NR3C2*])^1,2^. Thus, the SR is composed of two parts: the central perception of the stimulus and production of the effector molecule (GCs), and the physiological changes occurring in diverse tissues by the function of the GCs and their receptor binding^3^. Behavioral changes occur together with these physiological changes. The responses from the brain and peripheral tissues are intertwined and modulate each other’s states^4^.

Despite the connotation of the name, the SR machinery operates in both stressful (phasic) and basal (tonic) states of the body^3,5^. During the phasic response after encountering potent stressors, high levels of GCs are secreted and drive a broad range of physiological and behavioral changes. Increased lipolysis and blood glucose levels, increased attention to the stressor, and increased or halted locomotion are some examples^6,7^. At the basal level, GCs are secreted in a pulsatile ultradian pattern via the same HPA axis^1,8^ and (among the many functions) synchronize the circadian cycles between the central clock of the suprachiasmatic nucleus (SCN) and the clocks of the peripheral tissues in the rest of the body^9–12^. In this manner, the SR machinery and GCs regulate basal wakefulness and metabolism^13^. Based on the time of the day and states of the body, GCs constitutively modulate the expression levels of many genes in diverse tissues^12,14–18^.

Such broad-scale actions are made possible by the biphasic (slow or rapid) signaling of the GCs and its receptor (GR). While glucocorticoid receptor (GR) is a transcription factor that modulates gene expression levels (slow, genomic response), GR also functions as a signaling molecule enabling a rapid, non-genomic GC response within seconds to minutes of a stimulus exposure^3,19^. The long-lasting modulation of gene expression and rapid signaling in both the stressful (phasic) and basal (tonic) states make GCs the key molecule with which our perception in the brain is translated into tangible physiological and behavioral changes. Together with the nervous system, GCs impart our body’s physiological responses and coordinate appropriate adaptations in accordance with the changing environment, both gradually and rapidly^7,20^. Thus, it is not a surprise that altered HPA axis activity is one of the most common findings in people with psychiatric disorders. Peripheral physiological symptoms accompany mental burdens in mood disorders and the ways in which psychological and somatic symptoms influence each other continue to be of importance^21,22^. Thus, we sought to deepen our understanding of the dynamics and role of key HPA axis receptors during an adaptive process to changing environmental conditions. Such knowledge may bring insight into how the HPA axis operates and how maladaptation of our body to the environmental challenges may arise. This context is important for interpretation of behavioral data when using zebrafish to understand gene environment interactions that are critical for proper function of vertebrate stress response and ultimately human health.

The zebrafish is well-suited for such investigations involving the SR and corresponding changes in physiology and behavior. The hypothalamic-pituitary-interrenal (HPI) axis in zebrafish is functionally homologous to the mammalian HPA axis^23,24^. The genes relevant to HPI axis functions are expressed around the time of hatching (2.5 days post-fertilization [dpf]), and the HPI axis responds to exogenous stressors from 3 to 4 dpf onward^24–29^. Zebrafish are a diurnal species with similar circadian pattern as humans^30^. Larval zebrafish develop rapidly, establishing most organ systems by 5 dpf, including the visual system^25,31^. Larval zebrafish exhibit a repertoire of phototaxis and prototypical swim behavior^32,33^. Larvae (5 dpf) prefer mildly lit environments, swimming toward a lit spot if not overly bright, and search for light when placed in darkness (positive phototaxis)^33–35^. Using a larval zebrafish model, we specifically asked what the role of the key HPI axis receptors is during the adaptation to changing ambient light conditions. We did not presume that the change itself is a stressful encounter, rather inquired the role of the HPI axis during the period of changing illumination. We leveraged a photo adaptive behavior in larval zebrafish that we previously showed to be dependent on key HPI axis receptors. Dark-acclimated zebrafish larvae moved significantly more after a 1-min illumination of white light in the post-illumination darkness, which was dependent on the key receptors in the HPI axis^23^. With zebrafish that carry a mutation in *mc2r* (ACTH receptor), *nr3c1* (glucocorticoid receptor), or *nr3c2* (mineralocorticoid receptor), we had shown that *mc2r* and *nr3c1* homozygous (HM) mutants had decreased locomotion in the post-illumination darkness after 1-min illumination, whereas *nr3c2* mutants did not^23^. Using the same mutant zebrafish lines, we expanded our investigation into locomotor responses to changing ambient illumination.

**Figure 1:**
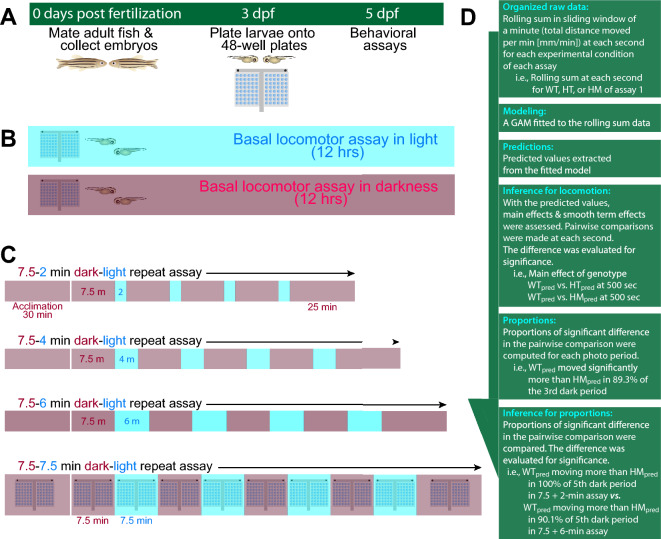
Schematics of experimental process and behavioral assay paradigms. (**A**) Experimental flow. Embryos obtained on 0 days-post-fertilization (dpf) through natural spawning. Healthy larvae were plated onto a pair of 48-well plates on 3–4 dpf. Assays were performed on 5 dpf unless otherwise stated. (**B**) Baseline assays. Larvae were videorecorded without any exogenous stimuli for about 12 h between 9:30 a.m. and 10:30 p.m. in light or darkness. (**C**) Dark–light repeat assays. Fish were observed in a changing illumination regimen i.e., 30-min acclimation + 4 × [7.5-min dark + 7.5-min light] + 25-min dark. Some assays do not include the final [25-min dark] component since they were performed before the experimental protocol was established. (**D**) Statistical analysis pipeline post-experiment. GAM modeling produces main effects of the predictors (i.e., genotype, illumination, developmental stages). When the main effect of the predictor is statistically significant, pairwise post-hoc analyses were conducted to identify the significant difference temporally (time points) and in the comparison group. The outcome of the statistical significance in the time window was summarized into proportions of significance over the entire range of time. Subsequently, the proportion was assessed for statistical significance to obtain the difference between different assay regimens (i.e., [7.5 + 2-min] assay vs. [7.5 + 7.5-min] assay). Inferences for the proportions were not made for baseline assays because there was only one assay regimen for the baseline assay (continuous recording in a dark or lit condition). GAM: generalized additive model.

We hypothesized that fish with HPI axis perturbation show compromised light adaptation. In the expanded paradigm, we provided alternating illumination and dark conditions. Fish were acclimated in darkness, and dark and light conditions were provided for 7.5 min in four cycles finishing the experiment in 25 min of darkness: dark acclimation (30 min) + 4 × [dark (7.5 min) + light (7.5 min)] + dark (25 min) (Fig. 1). We observed changes in locomotor responses after changing the duration of illumination to 2, 4, or 6 min (i.e., dark acclimation (30 min) + 4 × [dark (7.5 min) + light (2 min)] + dark (25 min). When we varied the illumination in duration and intensity, HPI axis receptors were required to mount increased locomotor response only after shorter durations of light (2 and 4 min), but not after longer illumination (7.5 min). At baseline without any changes in illumination, *nr3c1* mutant larvae moved less than their WT siblings in constantly lit and dark conditions. Dimmer and brighter light elicited the same pattern of response while dimmer light did not require HPI axis signaling to do so even during shorter durations of light. Using the adaptive behavior of zebrafish, we report that functioning HPI axis receptors (*mc2r*, *nr3c1*) are required to facilitate photoadaptation, enabling adaptation even after a short 1 or 2-min illumination, and to sustain basal locomotor activity. Rejecting our initial hypothesis, HPI axis perturbation did not lead to a uniform disruption of light adaptation process. When the stimulus was long enough in duration (≥ 6 min) or was low intensity (300 lx), the adaptive behavior could be achieved without HPI axis activity, indicating multiple pathways that lead to the adaptation. The HPI axis contributes to characteristic locomotor responses during light adaptation at various stages, facilitating initiation and maintaining higher levels of locomotion following basal adaptation.

## Materials and methods

### Materials and equipment

We listed the materials and equipment used in this study (Supplementary Table S1).

### Zebrafish husbandry

Wild-type (WT) Mayo Recessive Free (MRF) zebrafish (*Danio rerio*) were used for all experiments. MRF zebrafish were originally purchased from Segrest Farm in Florida (~ 2010) and have been systematically outbred to maintain a genetically diverse, healthy stock^36^. Fish were handled and cared for following standard practices^37^. The Institutional Animal Care and Use Committee (IACUC) in the Mayo Clinic (A345-13-R16, A8815-15) approved the animal husbandry and study protocol. All experiments and methodologies were performed in accordance with relevant guidelines and regulations. The “Methods” and “Results” are reported in accordance with ARRIVE guidelines^38^. Adult fish were kept in a 9 L (25–30 fish) or 3 L (10–15) housing tank at 28.5 °C with a light:dark (14:10) cycle. Light is on at 8:30 A.M. All experiments in this study were conducted between 4 and 7 dpf before the zebrafish sex is determined at about 15 dpf^31,39–41^, and thus sex determination was not made.

### Mutant zebrafish lines

The same WT and TALEN (transcription activator-like effector nuclease)-induced mutant zebrafish lines that we previously reported were used^23^. All fish were maintained through outbreeding. There were three *mc2r* mutant lines that each carried a frameshift mutation in exon 1 (two 4- and one 5-base pair deletions; *mn*^*57*^, *mn*^*58*^, and *mn*^*59*^, respectively). The annotation on the gene *mc2r* in the National Center for Biotechnology Information (NCBI) has changed from having 2 exons to having 1 exon since our previous report. Four *nr3c1* frameshift mutants were used, each of which carried a 7- or a 17-bp deletion in exon 2 (*mn*^*61*^, *mn*^*62*^) or a 4- or a 5-bp deletion in exon 5 (*mn*^*63*^, *mn*^*65*^). One *nr3c2* frameshift mutant was used that carried a 55-bp deletion in exon 2 (*mn*^*67*^). For detailed information on the mutant lines (i.e., the sequence of mutant alleles), refer to our previous paper^23^.

### Behavioral assay preparation

Adult mating pairs were placed in mating tanks separated by dividers (− 1 dpf). On the following day, the divider was pulled between 8:30 and 9:00 A.M. and embryos were obtained via natural spawning (0 dpf). Unfertilized and unhealthy embryos were cleaned up on 0, 1, and 3 dpf in the petri dish. Morphological defects (deformity, death) were the only exclusion criteria for an embryo to be excluded from an experiment. Larvae from different parent pairs were mixed in each petri dish to randomize the animals. On 3–4 dpf, a single larva was placed in each well of a 48-well plate. Larvae were caught from varying regions of the dish (i.e., center of the dish, areas close to the wall), from various depths (i.e., surface of the water, close to bottom of the dish), and throughout various swim behavior (i.e., fast swimmer, quiescent sitter) to ensure each 48-well plate contains larvae with diverse behavioral patterns. On 5–7 dpf, behavioral assays were performed in a custom-built assay chamber (Fig. 1A). From 0 to 7 dpf, plates were stored in an incubator with a light:dark cycle (14:10 h) at 28.5 °C.

### Custom light boxes

Light boxes were custom produced by the Mayo Clinic Division of Engineering. The light box has a control panel with two knobs that enable light intensity adjustment. Both infrared (IR) and white light have a low, medium, and high intensity. The illumination was provided from the bottom of the box through an additional translucent white acrylic board to evenly diffuse the light.

The spectral range of white light was approximately between 420 and 780 nm (STS-VIS; Ocean Optics Inc.; Supplementary Figs. S1–S3) and its light power (irradiance; wattage/unit area) was 20.5, 240.0, and 469.4 µW cm^−2^ for the low, med, and high, respectively (Benchtop optical power meter; 1936-R; Newport Corp.). When translated into brightness (illuminance; lux) measured by a light meter application on a cell phone, the power measurements were equivalent to ~ 300, 4000, and 8000 lx, respectively.

The spectral range of infrared (IR) light was approximately between 780 and 880 nm (STS-NIR; Ocean Optics Inc.; Supplementary Figs. S4–S6) and its light power was 6.3, 58.1, and 116.0 µW cm^−2^ for the low, med, and high, respectively. The IR light produces 0 lx in brightness measurement. For detailed information on the custom light boxes (e.g., dimensions), refer to our previous paper^23^.

### Basal/baseline locomotor activity assays

On the assay day, larvae were acclimated in either dark or light for 30 min in accordance with the recording condition before videorecording started (HDR-CX560V; Sony Corp.; Supplementary Table S1). An assay started around 9 am and was videorecorded for 13 h without any changes in the light condition (either dark or light continually; Fig. 1B) within the day light period of 14 h of the 14:10 (light:dark) cycle. Either white (469.4 µW cm^−2^; 8000 lx) or IR (116.0 µW cm^−2^; 0 lx) illumination was provided at the high intensity condition for an assay. Larvae were tested at 4, 5, 6, and 7 dpf.

### Dark–light repeat assays

All assays were performed at the high intensity for both IR (116.0 µW cm^−2^; 0 lx) and white (469.4 µW cm^−2^; 8000 lx) light, except the dim light assays. The dim light assays were done at the high intensity for infrared and at the low (20.5 µW cm^−2^; 300 lx) for white light.

The dark period was recorded in IR. Although zebrafish are thought to be unable to detect infrared and the acclimation to IR led to quiescence in locomotion, negative phototaxis to near-IR was reported^42^. The larval zebrafish on 5 dpf were acclimated in IR for 30 min and underwent the dark (7.5 min) and light (7.5 min) periods four times. In the assays with shorter durations of illumination, 2, 4, or 6-min illumination was provided while keeping the length of the dark phase constant at 7.5 min. Assays ended with a 25-min dark period. The regimens were abbreviated with the repeat element (i.e., [7.5 + 2-min]) in the text. Regimen: 30-min dark acclimation + 4 × [7.5-min dark + 7.5-min light] + 25-min dark (Fig. 1C). In some assays that were performed earlier in the project, the final 25-min dark period was omitted since the full experimental protocol was not established.

### Dim light assays

In the dim light assays, the same protocol was followed except that the lowest of three intensity settings was used for white light (20.5 µW cm^−2^; 300 lx)^23^.

### Statistical analysis

#### Explanatory (independent) variables

WT baseline assays: Developmental stage (4, 5, 6, and 7 dpf), illumination (dark and light), and time (videorecorded assay period. 1–43,200 s [12 h]; analyzed one data point per 30 s). *nr3c1* baseline assays: same as WT baseline assays, except that genotype (WT, heterozygous [HT], and homozygous [HM]) was an explanatory variable instead of the developmental stage. Dark–light repeat assays: genotype (WT, HT, and HM) and time (videorecorded assay period. 1–3600 secs [1 h]; one data point per second). The experimenters were blind to the genotype of fish since larvae were genotyped post experiments.

#### Response (dependent) variable

Locomotor activity was videorecorded (30 fps), and one data point was used for every second. Total distance moved for one minute is summed at every second in a sliding window of a minute (rolling sum; mm/min). The mean of the total distance moved per minute at each second was calculated for each experimental condition of the explanatory variable for each assay (i.e., WT, HT, and HM in assay 1). This rolling sum in each assay was the primary analysis unit. Since the variability in the dark–light assay is not established, we used our previous study to estimate the appropriate number of animals necessary. In our previous paper^23^, we used 500–1000 fish for a set of an experiment (i.e., 1-min light assay with *mc2r* siblings). We used a pair of 48-well plates and thus 500–1000 fish translate to approximately 5–10 assays. Our aim was to fit the number of assays between 5 and 10. However, since we intended to perform a set of experiment on multiple days to account for unexpected effects of a particular day and used natural spawning, the final number of assays in each set of experiment varied between 4 and 11, with the exception of dim light assays using WT fish. The dim light assay with WT fish was concluded with 3 assays per experiment.

#### Statistical analysis workflow

Using raw movement data obtained in csv files for each assay (Fig. 1D): (1) The total distance moved for a min (rolling sum) at each second was computed for each explanatory variable group, (2) a Generalized Additive Model (GAM)^43^ was fitted to the data and analyzed the main effect of each predictors (i.e., genotype, illumination, developmental stages), (3) pairwise comparison on the predicted response variable from the model was conducted at each second, (4) proportion of the significance during a photo period was computed, and (5) comparison between the proportions of the significance was performed among different assay paradigms. All analyses were performed using the R language (4.3.2; Eye Holes)^44^ and all figures except the schematics (Fig. 1) were produced with R. All primary data and supplementary materials were deposited in the open access data repository (Enter “10.6084/m9.figshare.25467865” on the figshare.com website). The core R scripts for GAM modeling were deposited to GitHub.com (https://github.com/moonlarkalto/HPA_gam) and the data sets for test-running the R scripts were deposited to FigShare at the same link).

#### Model

A generalized additive model (GAM) was developed to describe the locomotion of larval zebrafish in response to illumination changes. The generic formula for GAMs can be conceptually represented as:

1 $$g\left(E\left(Y\right)\right)={A}_{i}\theta +{f}_{1}\left({x}_{1i}\right)+{f}_{2}\left({x}_{2i}\right)+\dots +{\epsilon }_{i}$$

where $g$ is a link function, $E\left(Y\right)$ is mean (expected) values of the response variable, ${A}_{i}$ and $\theta$ are a design matrix and its parameter vector, respectively, of the linear term of ${A}_{i}\theta$, ${f}_{1}$ is a group of smooth functions to the term of ${x}_{1i}$, ${f}_{2}$ is a set of smooth functions to another explanatory variable of ${x}_{2i}$, and ${\epsilon }_{i}$ is an error term. The response variable $E\left(Y\right)$ can come from any exponential family and some non-exponential family distributions.

A GAM with the explanatory variables of genotype and time was fitted to the dark–light repeat assay data in R^44^ using the *mgcv* package (v1.8–42):^45^

2 $$\mathrm{rsums }\sim \mathrm{ geno }+{\text{s}}\left({\text{Time}},\mathrm{ k}=130,\mathrm{ by}={\text{geno}}\right)+{\text{s}}\left({\text{anid}},\mathrm{ bs}={\text{re}}\right)$$

where $rsums$ is the response variable of the rolling sum, $\sim$ denotes the relationship between the response and explanatory variables, $geno$ is a linear explanatory term as a categorical variable (WT, HT, and HM; the main effect of this term is estimated), $s$ constructs a set of smooth functions for the smooth term $Time$, $k$ (basis dimension) is the number of base functions that constitute the smooth functions for the term (sets the upper limit on the degrees of freedom for the $s$ smoother of the term), and $by$ means a separate set of smooth functions are estimated for the term $Time$ for each condition in $geno$. The smooth term for $anid$ (representing each assay) is added as a random effect among the different assays where $bs$ is an option to choose the type of spline (the thin plate spline [$tp$] is used as the default technique to produce smooth curves) and $re$ is a spline option of random effect for the term $anid$. Refer to the *mgcv* package manual for information^46^.

Similarly, a GAM with the explanatory variables of illumination condition and developmental stage was fitted to the baseline assay data. There was no significant interaction detected among the terms, so the interaction was not modeled.

3 $$\mathrm{rsums }\sim \mathrm{ illu}+{\text{dev}}+{\text{s}}\left({\text{Time}},\mathrm{ k}=220,\mathrm{ by}={\text{illu}}\right)+{\text{s}}\left({\text{Time}},\mathrm{ k}=220,\mathrm{ by}={\text{dev}}\right)+{\text{s}}\left({\text{anid}},\mathrm{ bs}={\text{re}}\right)$$

When the experimental period is longer (about an hour in the dark–light repeat assay vs. about 12 h in the baseline assay), the number of base functions necessary to fit the model increased ($k=130$ in the Eq. (2) vs. $k=220$ in Eq. (3)). In GAMs, increased numbers of base functions produce more wiggly lines, more closely reflecting the changes in the actual data, while a straight line is produced when $k=1$. To prevent overfitting, unnecessarily high $k$ values were penalized during the fitting process as well as when competing models were compared for fitness. The experimenter only sets the upper limit ($k$) and the optimal $k$ value is determined by the underlying optimization algorithm in the *mgcv* package, which can be further validated for its fitness statistics.

The model was first heuristically evaluated by the proportion of the variation explained, the distribution of the residuals, and the parameters produced by the $gam.check$ function in *mgcv*. The model was considered inadequate when (1) the predicted values do not reasonably trace the actual data upon visual inspection, (2) the proportion of variation explained is too low, (3) the *p*-value (different from the *p*-value for inference on main effects) for each smooth term in the $gam.check$ summary is too small (has to be comfortably *not significant*). Among the valid models with differing parameters, the fitness of those models was compared using Akaike information criterion (AIC).

#### Inference for the locomotion

Once a GAM model is fitted, the overall effects (main effects) and significance of each linear and smooth term on the response variable were evaluated, visualized, and recorded. Based on the fitted model, the predicted values of the response variables were extracted using the $predict$ function. With the predicted values of the rolling sum, post hoc pairwise comparisons were performed using the *emmeans* package (v1.8.5)^47^. The estimated marginal means (EMMs; least-squares means) for each explanatory variable condition was computed (i.e., Eq. (4)), the difference between a pair of conditions was analyzed, and the significance of the difference was produced at every second (i.e., Eq. (5)):

4 $${\text{emmeans}}({\text{fishmodel}}, \sim {\text{geno}},{\text{at}}={\text{list}}({\text{Time}}=500)$$

5 $${\text{pairs}}({\text{emmeans}}({\text{fishmodel}}, \sim {\text{geno}},{\text{at}}={\text{list}}\left({\text{Time}}=500\right))$$

The pairwise comparison ($pairs$) produced the Tukey-adjusted *p*-values based on the *t* ratios drawn from the Studentized Range distribution, adjusted for multiple comparisons. The *p*-values were used to determine whether there is a significant difference in the distance moved between the two conditions (i.e., WT vs. HM) at each second.

#### Proportion of significance during a photo period

Based on the inference (significance) drawn at every second, the proportion that a group moved significantly more than the comparison group during a given photo period was computed (i.e., *mc2r* WT larvae significantly moved more than their HM siblings in 89.3% of the “3rd Dark” photo period in the 7.5 + 2-min illumination assay; Supplementary Fig. S12; Supplementary Table S67; Fig. 4Ac).

#### Inference for the proportion

The proportions of significance during a photo period were compared using the two-proportions $z$ test with Yates’ continuity correction to see whether the durations of illumination led to difference in the proportions of significant difference in locomotion^48–50^ (i.e., *nr3c1*^*ex5*^ WT larvae moved significantly more than their HM siblings in 100% of the “5th Dark” photo period in the 7.5 + 2-min illumination assay, whereas the proportion of significant difference in the 7.5 + 6-min illumination assay was 90.1%. It was asked if these two proportions were significantly different; Supplementary Data S32; Supplementary Table S67; Supplementary Figs. S21 and S23).

## Results

### Generalized additive model describes locomotor response during environmental changes

To better understand the dynamics of locomotor response over time, a generalized additive model (GAM) was developed (Fig. 1D; “Model”, “Statistical analysis” in the “Methods”). A GAM for each assay consisted of the linear (parametric) and smooth terms that described the response variable, locomotion. The effect of each term on locomotion was evaluated for its main effect, which reports whether a predictor/explanatory variable had a statistically significant effect (i.e., genotype, illumination, developmental stage). With all the assays, the overall variation in locomotion explained by the model ranged from 68.3 to 97.5% (mean = 86.6%), demonstrating a satisfactory explanatory power (Supplementary Data S1–S23). The residuals, unexplained variations, and fitted values were visualized to assess their appropriateness (Supplementary Figs. S63–S85). Post hoc pairwise analysis was performed to assess the significance of the difference in locomotion between the groups every second (Supplementary Tables S2–S66). The proportions of the significance between different assay regimens were compared to understand the effect of different durations of illumination (i.e., 4 × [7.5 dark + 2-min light] regimen vs. 4 × [7.5 + 7.5-min] regimen; Supplementary Figs. S8–S62; Supplementary Data. S24–S59; Supplementary Tables S67–S70).

To make the analysis process more rigorous, we used the mean of each experimental group in each assay, rather than the raw movement measurement of individual fish conventionally utilized in zebrafish behavioral studies (“Response variable”, “Statistical analysis” in the “Methods”). The aggregation of the locomotor response mitigated the severe skewedness in the data.

### WT larvae move more in light than in darkness during the day

To understand the changes in locomotion following illumination changes, we first established the pattern of basal locomotor activity during the day without any stimuli. We were curious whether zebrafish would move more in a lit or dark condition in the daytime. As a diurnal species, zebrafish have a circadian cycle that has evolved for high activity during the day and rest at night^51^. In our previous studies, movement in our wild-type larval fish was greater when acclimated in lit conditions (e.g., salt assays) than in dark conditions (e.g., light assays)^23,52^. Indeed, others have observed larval fish move more in lit conditions^53^. We have previously demonstrated that activation of a transcriptional biosensor followed a baseline circadian rhythm and activation by exogenous cortisol was lost in TALEN-injected fish that targeted a locus in *nr3c1* exon 2^51^. Here, we intended to clearly establish if the baseline larval locomotion would follow the circadian rhythm regardless of the condition of current illumination at a moment or the condition of ambient light (lit or dark) would dictate the levels of locomotion overriding the circadian cycle (called “masking”). We recorded wild-type (WT) larvae (4-, 5-, and 6-days post-fertilization [dpf]) from around 9:30 A.M. to 10:30 P.M. in constant illumination or darkness.

**Figure 2:**
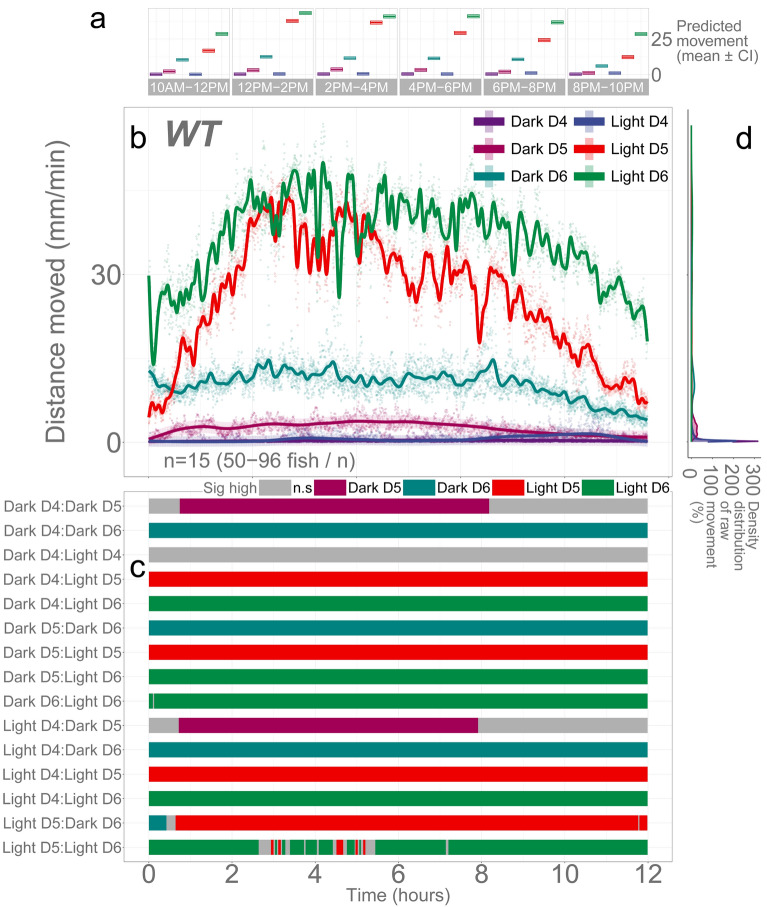
Illumination and developmental stages are determinants of basal locomotion in WT larvae. The x-axis of the panels (**a**–**c**) are Time in hours labeled below panel (**c**). The y-axis is given in each panel. (**a**) Descriptive statistics summarizing mean activity level. Locomotor activity (mean predicted value [mm/min] ± 95% CI) for each experimental condition predicted by the generalized additive model (GAM) for each time span. (**b**) Basal locomotor activity of WT larvae over 12 h. The scatterplot (points) shows actual mean locomotor activity (mm/min) for each experimental condition of each assay. The line graph shows predicted locomotor activity for each experimental condition by the GAM (predicted value ± 95% CI). (**c**) Time points where an experimental condition showed significantly higher locomotor activity compared to the other in a pairwise comparison. The color shown indicates the group with a significantly higher outcome in a pairwise comparison. For the actual numbers of pairwise comparison, refer to the corresponding Supplementary Tables of each Figure. For the main effects and smooth effects of the model, refer to the corresponding Supplementary Data for each Figure. (**d**) Histogram of individual fish locomotion (y-axis is the same as subpanel (**b**) [Distance moved]). Density distribution of actual mean locomotor activity shows severely right skewed distribution (low locomotor response). The integration of the curve equals 100%. Since individual y-axis bin (distance moved [mm/min]) is smaller than 1 (i.e., 0.2 mm/min), the x-axis values reach over 100% while the area under the curve is still 100%. (D: dark, D4: 4 dpf, L: light, n.s: not significant).

We found that illumination was a critical determinant for the larvae’s baseline locomotion on days 5 and 6 but not on day 4 (Fig. 2; Supplementary Data S1). Light significantly increased locomotion in both 5 and 6 dpf fish (light, main effect, *p* = 0.0026 and *p* < 2e − 16, respectively; Supplementary Data S1, Supplementary Figs. S8 and S9; all *p*-values provided in Supplementary Tables S3 and S4 and all proportions provided in Supplementary Tables S67–S68). However, there was no difference in 4 dpf fish (light, main effect, p = 0.6735). The developmental stage was another important determinant for basal locomotion. Compared to 4-dpf larvae, 5- and 6-dpf larvae moved significantly more in the corresponding light conditions (4 dpf vs. 5 and 6-dpf in either dark or light). A dark environment decreased baseline activity, masking the basal circadian activity levels in light during the day in larvae aged between 5 and 6 days. Since the overall activity levels were low in 4-dpf fish, there was barely any difference in baseline locomotion in 4-dpf larvae.

### *nr3c1* mutant larvae move less than WT siblings in light and darkness during the day

**Figure 3:**
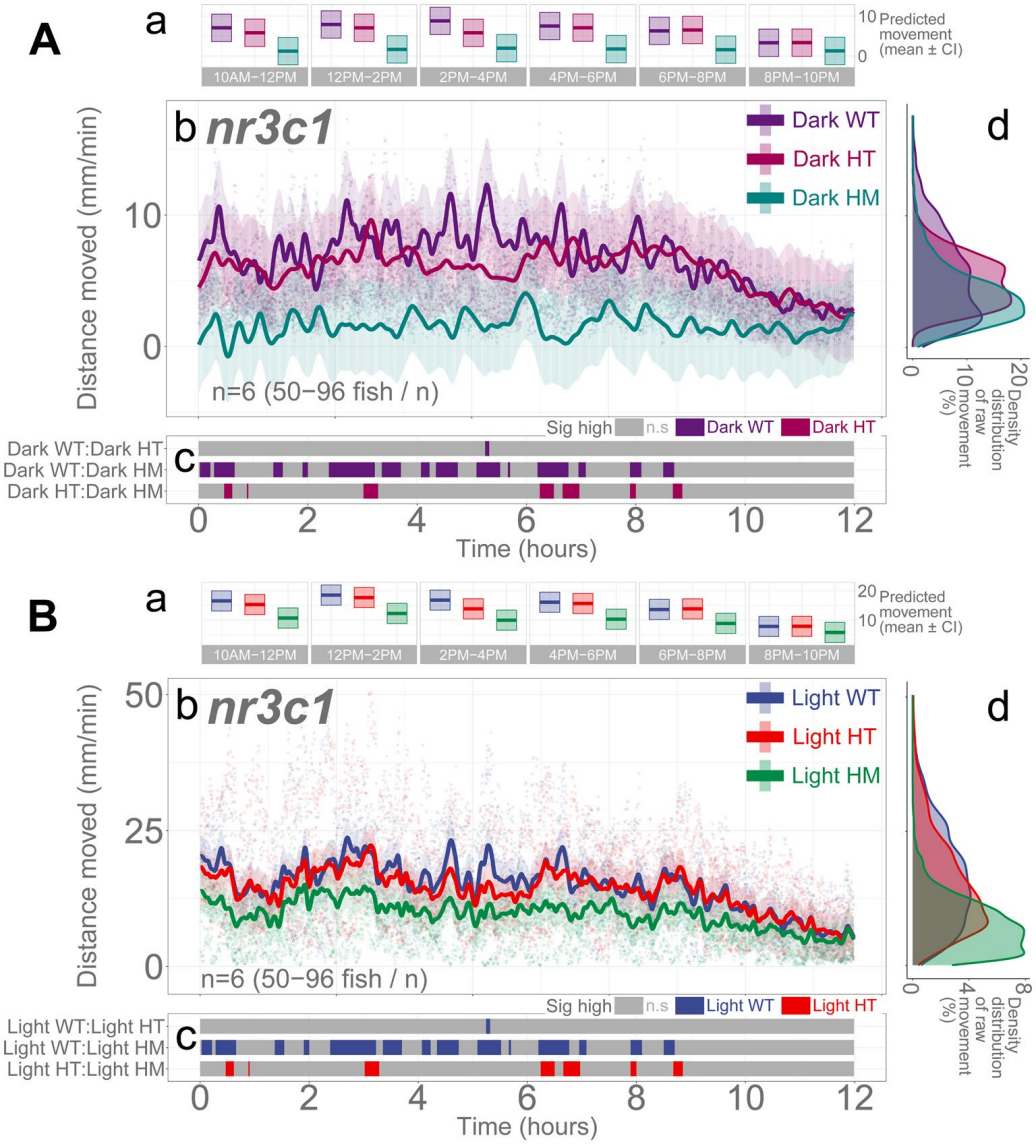
Illumination and genotype are determinants of basal locomotion in *nr3c1* knockout larvae. Aa Ba Locomotor activity (mean predicted value [mm/min] ± 95%CI) for each genotype and experimental condition ((**A**) in darkness, (**B**) in light). (**Ab, Bb**) Basal locomotor activity of *nr3c1* knockout larvae over 12 h. The scatterplot (points) shows actual mean locomotor activity (mm/min) for each experimental condition of each assay. The line graph shows predicted locomotor activity for each experimental condition by the GAM (predicted value ± 95%CI). (**Ac, Bc**) Time points where an experimental condition showed significantly higher locomotor activity compared to the other in a pairwise comparison. The color shown indicates the group with a significantly higher outcome in a pairwise comparison. (**Ad, Bd**) Histogram of individual fish locomotion (y-axis is the same as subpanel [**b**]). Density distribution of actual mean locomotor activity shows severely right skewed distribution (low locomotor response). The integration of the curve equals 100%. (D: dark, L: light, WT: wildtype, HT: heterozygous, HM: homozygous, n.s: not significant, *nr3c1* genotypes: *nr3c1*^+*/*+^ [WT], *nr3c1*^+*/mn63*^ [HT], *nr3c1*^*mn63/mn63*^ [HM, KO], or *nr3c1*^+*/mn65*^ [HT], *nr3c1*^*mn65/mn65*^ [HM, KO]).

Following the study of basal locomotion in WT fish, we investigated the basal locomotion of the *nr3c1* fish (5 dpf) in which glucocorticoid receptor (*nr3c1*) is knocked out in homozygous (HM) larvae (*nr3c1*^*mn63/mn63*^ or *nr3c1*^*mn65/mn65*^)^23^. Similar to the WT stock fish, illumination was a critical determinant of locomotor activity of *nr3c1* KO fish. Compared to darkness, a lit condition increased locomotion (light; main effect, *p* = 3.27e − 6; Supplementary Data S2; Fig. 3). Genotype was another determinant. *nr3c1* WT and heterozygous (HT; *nr3c1*^+*/mn63*^, *nr3c1*^+*/mn65*^) siblings showed comparable levels of locomotion in darkness or light (HT, main effect, *p* = 0.683). Homozygosity (*nr3c1*^*mn63/mn63*^ or *nr3c1*^*mn65/mn65*^) decreased overall locomotion (HM, main effect, *p* = 0.014). WT larvae moved significantly more than HM siblings in 34.84% of the time both in darkness and illumination, and the significant difference was mainly found before 7 P.M. (Fig. 3Ac,Bc); Supplementary Figs. S10 and S11; Supplementary Tables S6 and S7 and Tables S67–S68).

### *mc2r* mutant larvae show less activity than WT siblings in darkness only when illumination duration is shorter

After establishing that both darkness and blocking an HPI axis receptor (*nr3c1*) activity decrease basal locomotion in larvae, we investigated the dynamic changes in locomotion in response to changing illumination. We previously showed that, to increase locomotor activity in post-light darkness after a brief 1-min illumination, functioning HPI axis receptors are essential as loss of *mc2r* and *nr3c1* significantly decreased the locomotor response in these fish^23^. Our initial hypothesis on the larval response in the dark–light repeat assay was that fish with a mutation in one of the key HPI axis receptor genes would have a deficiency in mounting an appropriate locomotor response to changing light. However, the duration of illumination is a determinant of the ensuing locomotor activity in darkness, as well as the HPI axis genes.

**Figure 4:**
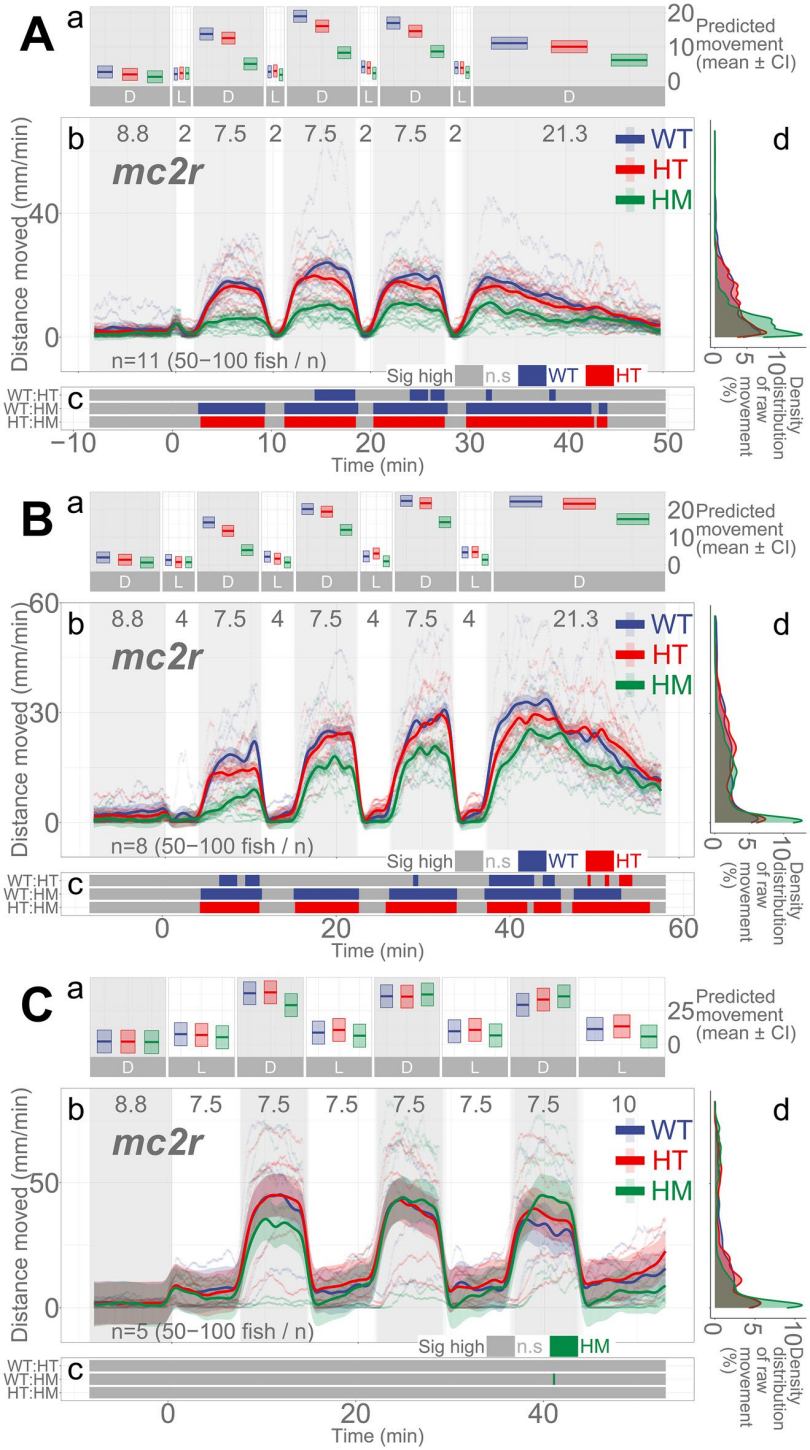
*mc2r* knockout larvae respond differentially in darkness based on the durations of illumination and genotype. (**Aa**, **Ba**, **Ca**) Locomotor activity (mean predicted value [mm/min] ± 95% CI) for each experimental condition predicted by the GAM for each photo period (gray: dark, white: light period). (**Ab**, **Bb**, **Cb**) Locomotor response of *mc2r*^*ex1*^ larvae during dark–light repeat assays. The scatterplot (points) shows actual mean locomotor activity (mm/min) for each experimental condition of each assay. The line graph shows predicted locomotor activity for each experimental condition by the GAM (predicted value ± 95% CI; gray: dark, white: light period). (**Ac**, **Bc**, **Cc**) Time points where an experimental condition showed significantly high locomotor activity compared to the other in a pairwise comparison. The color shown indicates the group with a significantly higher outcome in a pairwise comparison. (**Ad**, **Bd**, **Cd**) Histogram of individual fish locomotion (y-axis is the same as subpanel [**b**]). Actual mean locomotor activity shows severely right skewed distribution (low locomotor response). The integration of the curve equals 100%. (D: dark, L: light, WT: wildtype, HT: heterozygous, HM: homozygous, n.s: not significant, *mc2r* genotypes: *mc2r*^+*/*+^ [WT], *mc2r*^+*/mn57*^ [HT], *mc2r*^*mn57/mn57*^ [HM, KO], *mc2r*^+*/mn58*^ [HT], *mc2r*^*mn58/mn58*^ [HM, KO], or *mc2r*^+*/mn59*^ [HT], *mc2r*^*mn59/mn59*^ [HM, KO]).

The ACTH receptor (*mc2r*) on the adrenal gland (interrenal cells in zebrafish) is a key HPA/I axis receptor that initiates glucocorticoid synthesis. When *mc2r* was knocked out (*mc2r*^*mn57/mn57*^*, mc2r*^*mn58/mn58*^*,* or *mc2r*^*mn59/mn59*^), the overall locomotion in darkness was significantly less than that of WT siblings when repeated illumination was 2 min (HM, main effect, *p* = 8.007e − 6) and 4 min (main effect, *p* = 0.00015), but not with 7.5-min illumination (main effect, *p* = 0.758; Fig. 4; Supplementary Data S3–S5). The proportion of locomotion in which WT larvae moved significantly more than their HM siblings in darkness (the 3rd, 4th, and 5th dark periods) was 93.9, 96.34, and 0% in the 2-, 4-, and 7.5-min light repeat assays, respectively (Supplementary Figs. S15–S17; Supplementary Table S69). The difference in proportions was significant between the 2- and 4-min light repeat assays (two-proportions *z* test, ${\chi }^{2}=8.243$, *p* = 0.004; Supplementary Data S25), the 2- and 7.5-min light repeat assays (${\chi }^{2}=2429.0$, $p\approx 0$; Supplementary Data S27), and the 4- and 7.5-min light repeat assays (${\chi }^{2}=2555.1$, *p* $\approx$ 0; Supplementary Data S29). Therefore, the *mc2r* HM larvae could mount an equivalent locomotion profile compared to WT siblings as the duration of illumination increased (7.5 min).

### *nr3c1* mutant larvae move less than WT siblings in darkness when illumination duration is shorter

**Figure 5:**
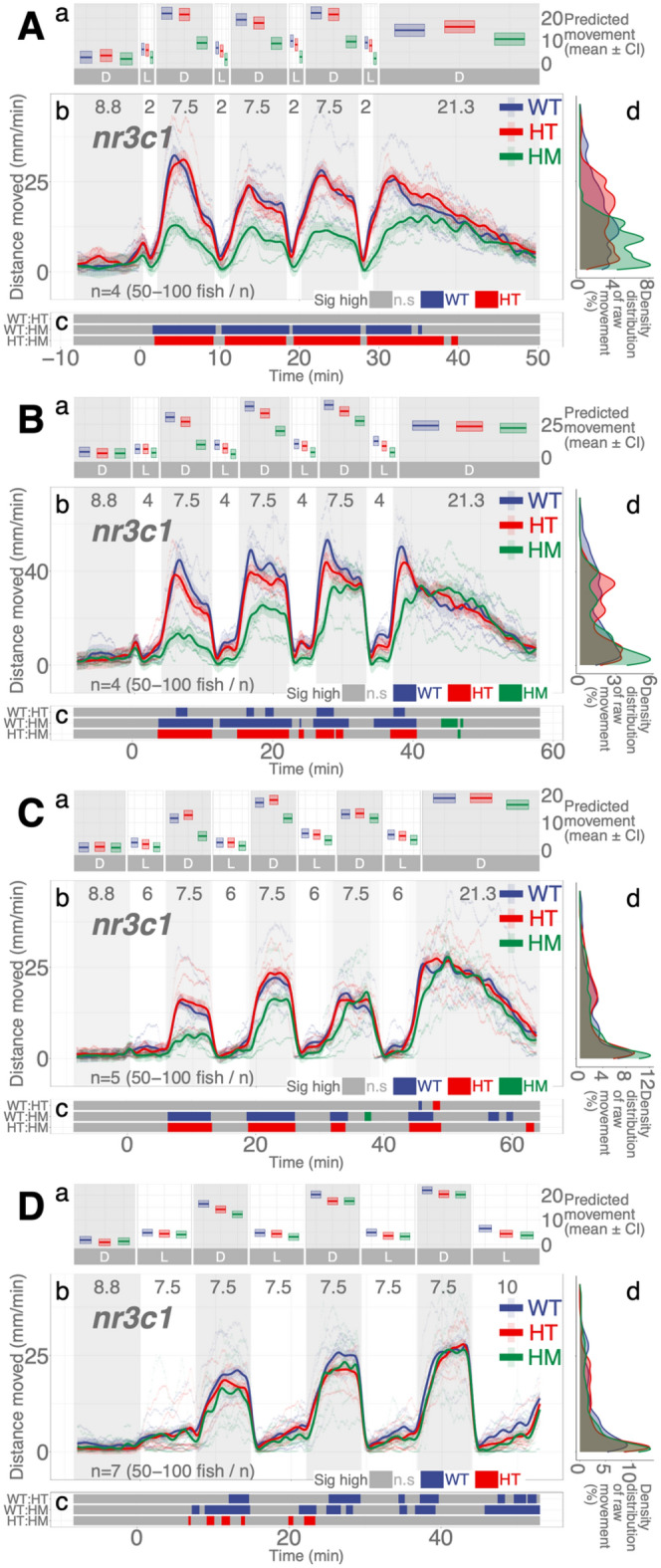
*nr3c1* knockout larvae respond differentially in darkness based on the durations of illumination and genotype. (**Aa**, **Ba**, **Ca**, **Da**) Locomotor activity (mean predicted value [mm/min] ± 95% CI) for each experimental condition predicted by the GAM for each photo period (gray: dark, white: light period). (**Ab**, **Bb**, **Cb**, **Db**) Locomotor response of *nr3c1* larvae during dark–light repeat assays. The scatterplot (points) shows actual mean locomotor activity (mm/min) for each experimental condition of each assay. The line graph shows predicted locomotor activity for each experimental condition by the GAM (predicted value ± 95% CI; gray: dark, white: light period). (**Ac**, **Bc**, **Cc**, **Dc**) Time points where an experimental condition showed significantly high locomotor activity compared to the other in a pairwise comparison. The color shown indicates the group with a significantly higher outcome in a pairwise comparison. (**Ad**, **Bd**, **Cd**, **Dd**) Histogram of individual fish locomotion (y-axis is the same as subpanel [**b**]). Density distribution of actual mean locomotor activity shows severely right skewed distribution (low locomotor response). The integration of the curve equals 100%. (D: dark, L: light, WT: wildtype, HT: heterozygous, HM: homozygous, n.s: not significant, *nr3c1* genotypes: *nr3c1*^+*/*+^ [WT], *nr3c1*^+*/mn63*^ [HT], *nr3c1*^*mn63/mn63*^ [HM, KO], or *nr3c1*^+*/mn65*^ [HT], *nr3c1*^*mn65/mn65*^ [HM, KO]).

Nr3c1 is the canonical glucocorticoid receptor that binds to glucocorticoids (i.e., cortisol, corticosterone) and drives the various stress responses in the central nervous system and peripheral tissues. Homozygous mutant larvae in *nr3c1*^*ex5*^ (*nr3c1*^*mn63/mn63*^ or *nr3c1*^*mn65/mn65*^) showed significantly less locomotion in darkness in the 2-, 4-, and 7.5-min repeat assays (HM, main effect, *p* = 0.00054; *p* = 0.0029, and *p* = 0.0465, respectively), but not in 6-min repeat assays (main effect, p = 0.057; Fig. 5; Supplementary Data S6–S9). The proportion of locomotion where the WT fish moved significantly more than their HM siblings in darkness was 100, 87.78, 68.1, and 60.88% when 2-, 4-, 6-, and 7.5-min illumination was repeated (Supplementary Figs. S25–S28; Supplementary Table S69). The difference in proportions was significant in all pairwise comparisons between 2- and 4-min (two-proportions *z* test, ${\chi }^{2}=171.48$, *p* = 3.510e − 39; Supplementary Data S31), 2- and 6-min (${\chi }^{2}=507.92$, *p* = 1.794e − 112; Supplementary Data S33), 2- and 7.5-min (${\chi }^{2}=647.77$, *p* = 6.828e − 143; Supplementary Data S35), 4- and 6-min (${\chi }^{2}=153.23$, *p* = 3.42e − 35; Supplementary Data S37), 4- and 7.5-min (${\chi }^{2}=253.14$, *p* = 5.373e − 57; Supplementary Data S39), and 6- and 7.5-min repeated illumination (${\chi }^{2}=15.632$, *p* = 7.694e − 5; Supplementary Data S41), showing that the proportion of the difference in locomotion between the WT and HM siblings changes as the illumination duration changes.

In another allele of *nr3c1* where the frameshift mutation was introduced in exon 2, the effect of the duration of illumination was clearer. Homozygous mutant larvae in *nr3c1*^*ex2*^ moved significantly less in darkness when repeated illumination was 4 min (HM, main effect, *p* = 0.0071), but not when illumination was 6 min (*p* = 0.197) and 7.5 min (*p* = 0.615; Supplementary Fig. 7; Supplementary Data S21–S23). The proportion of locomotion where the WT fish moved significantly more than their HM siblings in darkness was 94.65, 71.58, and 0% when the repeated illumination was 4, 6, and 7.5 min (Supplementary Figs. S57–S59; Supplementary Table S69). The difference in overall activity levels in darkness between the WT and HM siblings decreased in both *nr3c1*^*ex5*^ and *nr3c1*^*ex2*^ mutant fish (100, 87.78, 68.1, and 60.88%; Not determined, 94.65, 71.58, and 0% at 2, 4, 6, and 7.5 min), respectively, as the repeated illumination increased^54–56^, showing that the *nr3c1* HM mutants could mount increasingly similar locomotor responses in darkness as the duration of illumination increased.

### Mutations in *nr3c2* do not appear to have consistent effects on locomotion

**Figure 6:**
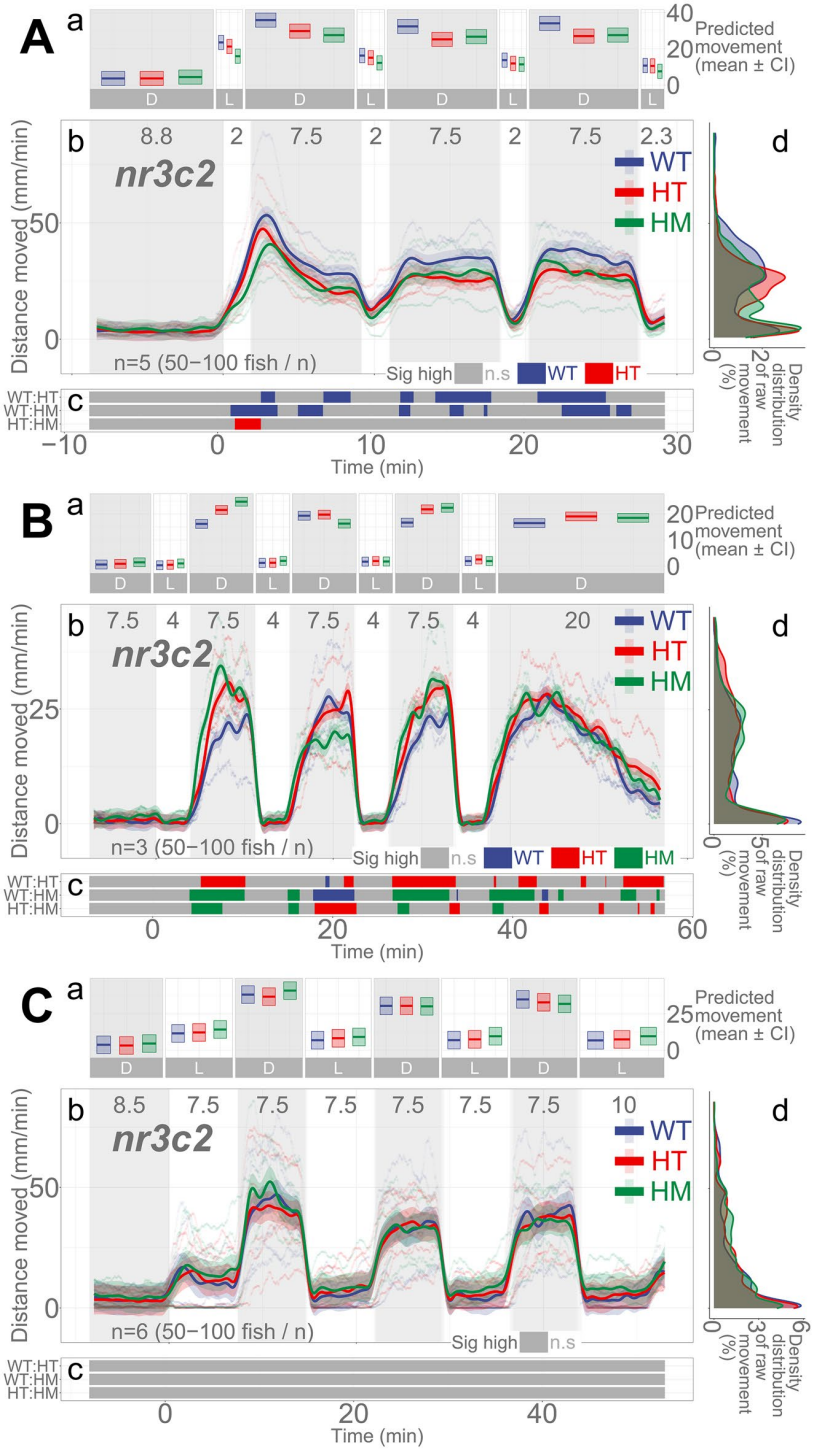
Mutation in *nr3c2* do not have main effect on locomotion. (**Aa**, **Ba**, **Ca**) Locomotor activity (mean predicted value [mm/min] ± 95% CI) for each experimental condition predicted by the GAM for each photo period (gray: dark, white: light period). (**Ab**, **Bb**, **Cb**) Locomotor response of *nr3c2*^*ex2*^ larvae during dark–light repeat assays. The scatterplot (points) shows actual mean locomotor activity (mm/min) for each experimental condition of each assay. The line graph shows predicted locomotor activity for each experimental condition by the GAM (predicted value ± 95% CI; gray: dark, white: light period). (**Ac**, **Bc**, **Cc**) Time points where an experimental condition showed significantly high locomotor activity compared to the other in a pairwise comparison. Despite such apparent significant difference at some time points, there was no main effect of being HM on locomotor response compared to WT siblings (Refer to the “Results”). The significance in the post-hoc analysis should not be taken into consideration. The tile graphs are provided to show the incoherent patterns in difference and for consistency with other Figures. (**Ad**, **Bd**, **Cd**) Histogram of individual fish locomotion (y-axis is the same as subpanel [**b**]). Density distribution of actual mean locomotor activity shows severely right skewed distribution (low locomotor response). The integration of the curve equals 100%. (D: dark, L: light, WT: wildtype, HT: heterozygous, HM: homozygous, n.s: not significant, *nr3c2* genotypes: *nr3c2*^+*/*+^ [WT], *nr3c2*^+*/mn67*^ [HT], *nr3c2*^*mn67/mn67*^ [HM, KO]).

Mineralocorticoid receptor (*nr3c2*) is another nuclear receptor that binds to glucocorticoids with higher affinity than does glucocorticoid receptor. However, we did not find a clear role in locomotor response to light changes in our previous paper^23^. Similarly, we could not identify any pattern of difference in locomotion between the WT and HM siblings (Fig. 6; *nr3c2*^+*/*+^ [WT], *nr3c2*^+*/mn67*^ [HT], *nr3c2*^*mn67/mn67*^ [HM, KO]). The HM mutant larvae in *nr3c2*^*ex2*^ apparently moved less in 2-min repeated illumination at some time points (Fig. 6Ac), but there was no main effect of homozygosity compared to WT siblings (HM, main effect, *p* = 0.104; Supplementary Data S10). Likewise, in 4- and 7.5-min repeated illumination, the HM larvae apparently moved more at some time points, but there was no main effect of homozygosity compared to WT siblings (*p* = 0.0728 and *p* = 0.758), respectively (Supplementary Data S11–S12). Since there was no main effect of homozygosity on locomotion, the meaning of the apparent decrease or increase of locomotion amongst HM siblings at some time points is unlikely to be biologically meaningful.

**Figure 7:**
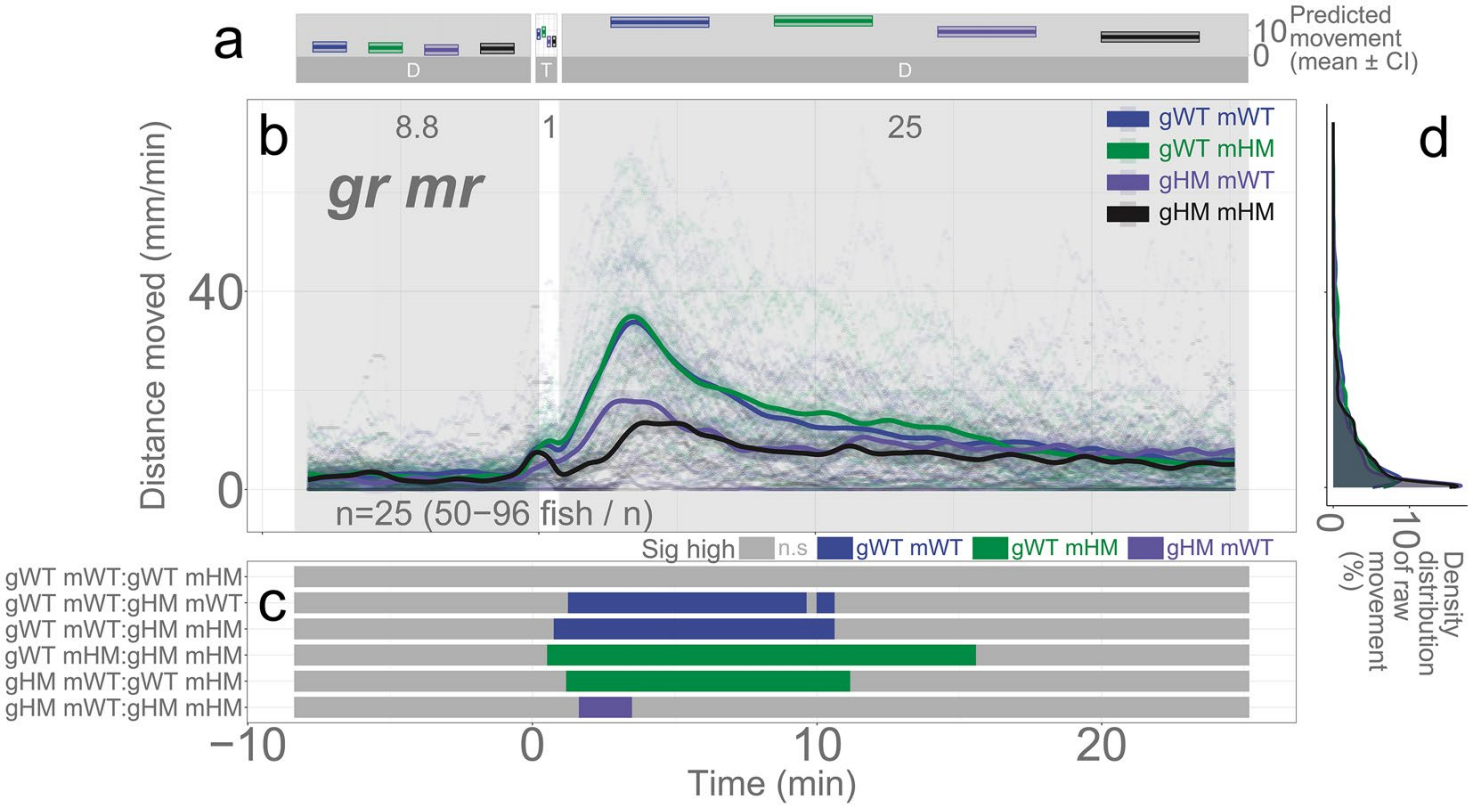
*nr3c1* (gr) and *nr3c2* (mr) double knockout larvae show varying locomotor activity levels in darkness after short illumination (1-min) (**a**) Locomotor activity (mean predicted value [mm/min] ± 95% CI) for each genotype (gr WT mr WT, gr WT mr HM, gr HM mr WT, gr HM mr HM), predicted by the GAM. Only WT and HM mutants are shown to increase readability in this figure while the full genotypic combinations are shown in Supplementary Fig. S87. The underlying data set is the same in the two figures. (**b**) Locomotor response in the baseline (dark) and post-illumination (dark). The scatterplot (points) shows actual mean locomotor activity (mm/min) for each experimental condition of each assay. The line graph shows predicted locomotor activity for each genotype by the GAM (predicted value ± 95% CI). (**c**) Time points where a genotype showed significantly high locomotor activity compared to the other in a pairwise comparison. (**d**) Density distribution of actual mean locomotor activity shows severely right skewed distribution. The integration of the curve equals 100% (gr: glucocorticoid receptor [*nr3c1*], mr: mineralocorticoid receptor [*nr3c2*], D: dark, T: treatment [illumination], WT: wildtype, HM: homozygous, n.s: not significant, *nr3c1* genotypes: *nr3c1*^+*/*+^ [WT], *nr3c1*^+*/mn63*^ [HT], *nr3c1*^*mn63/mn63*^ [HM, KO], or *nr3c1*^+*/mn65*^ [HT], *nr3c1*^*mn65/mn65*^ [HM, KO], *nr3c2* genotypes: *nr3c2*^+*/*+^ [WT], *nr3c2*^+*/mn67*^ [HT], *nr3c2*^*mn67/mn67*^ [HM, KO]).

In addition, we investigated the locomotor response of *nr3c1* and *nr3c2* double knockout fish after short (1-min) illumination to discern the differential contribution of *nr3c1* and *nr3c2* in the light responsive behavior. There was a significant effect of homozygosity in *nr3c1* mutants (*nr3c1*^*mn63/mn63*^; HM, main effect, p = 0.0337) whereas we did not find significance in *nr3c2* mutants (HM, main effects, p = 0.839; Fig. 6; Supplementary Data S60; Supplementary Tables S71, S72, and S73). Figure 7 only shows the WT and HM data of the double knockout fish to increase readability. For the full genotypic combinations, refer to Supplementary Fig. S87. Locomotor response is less in part of the time window in gr HM mr HM (*nr3c1*^*mn63/mn63*^*; nr3c2*^*mn67/mn67*^), compared to gr HM mr WT (*nr3c1*^*mn63/mn63*^*; nr3c2*^+/+^). However, the decreased locomotion was attributed more to the effect of *nr3c1* homozygosity (the overall decrease of locomotion in both gr HM mr WT and gr HM mr HM).

### Locomotor patterns over dark–light repeats are reproduced with much dimmer light

**Figure 8:**
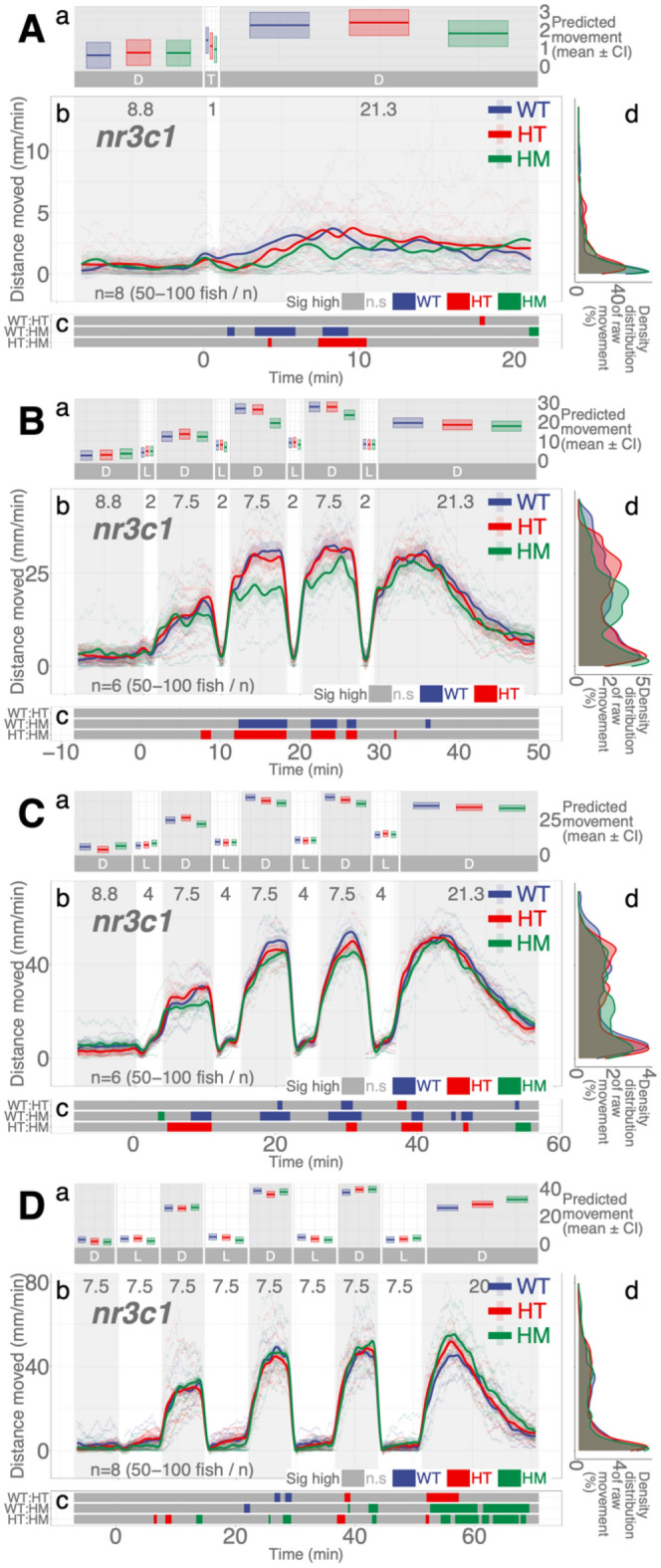
Much dimmer illumination reproduces the same pattern of dark–light responses in *nr3c1* knockout larvae and may lead to more effective photoadaptation. Dimmer illumination (20.5 µW cm^−2^; 300 lx) was used compared to that of all other experiments (469.4 µW cm^−2^; 8000 lx). IR illumination was the same (116.0 µW cm^−2^; 0 lx). (**Aa**, **Ba**, **Ca**, **Da**) Locomotor activity (mean predicted value (mm/min) ± 95% CI) for each experimental condition predicted by the GAM for each photo period (gray: dark, white: light period). A brief illumination assay (1-min light) without the repeat components was included to understand behavior in dim light (**A**). (**Ab**, **Bb**, **Cb**, **Db**) Locomotor response of *nr3c1*^*ex5*^ larvae during dark–light repeat assays. The scatterplot (points) shows actual mean locomotor activity (mm/min) for each experimental condition of each assay. The line graph shows predicted locomotor activity for each experimental condition by the GAM (predicted value ± 95% CI; gray: dark, white: light period). (**Ac**, **Bc**, **Cc**, **Dc**) Time points where an experimental condition showed significantly high locomotor activity compared to the other in a pairwise comparison. Despite such apparent significant difference at some time points, there was no main effect of being HM on locomotor response compared to WT siblings, starting from the 2-min light assays (Refer to the “Results”). The tile graphs are provided to show the patterns in difference. (**Ad**, **Bd**, **Cd**, **Dd**) Histogram of individual fish locomotion (y axis is the same as the subpanel b). Density distribution of actual mean locomotor activity shows right skewed distribution (low locomotor response). The integration of the curve equals 100%. (D: dark, L: light, WT: wildtype, HT: heterozygous, HM: homozygous, n.s: not significant, *nr3c1*^+*/*+^ [WT], *nr3c1*^+*/mn63*^ [HT], *nr3c1*^*mn63/mn63*^ [HM, KO], or *nr3c1*^+*/mn65*^ [HT], *nr3c1*^*mn65/mn65*^ [HM, KO]).

All light experiments were conducted with consistent illumination intensities: IR (116.0 µW cm^−2^; 0 lx) and white light (469.4 µW cm^−2^; 8000 lx). Since the duration of illumination (quantity of light by time) was a determinant of locomotor response in darkness, we asked whether lower intensities of illumination (quantity of light by intensity) would elicit the same behavioral adaptation and, if so, whether it would take longer. In the dim light assays, we used the same dark–light repeat regimen but used a much lower intensity of white light (20.5 µW cm^−2^; 300 lx). With dim light repeat assays, WT fish showed the same pattern of locomotor response as the assays with higher intensities of light (Supplementary Fig. S86), increasing locomotor response in darkness while having lower locomotion in light. With the *nr3c1*^*ex5*^ fish, the HM larvae (*nr3c1*^*mn63/mn63*^ or *nr3c1*^*mn65/mn65*^) showed equivalent levels of locomotion to WT fish in all 2-, 4-, and 7.5-min repeated dim illumination and there was no main effect of homozygosity on locomotion compared to WT siblings (HM, main effect, *p* = 0.224, *p* = 0.263, and *p* = 0.485), respectively (Supplementary Data S18–S20). Whereas such increased locomotor activity happened after longer illumination (> 6 min) with a brighter light condition, it occurred with shorter durations of light (2 or 4 min) in dimmer light. Since there was no main effect, further analysis was not pursued. The tile graphs (Fig. 8Bc,Cc,Dc) are provided to show the patterns in difference. Unexpectedly, dimmer illumination effectively facilitates the locomotor response in darkness after repeated illumination without a requirement for HPI axis signaling.

## Discussion

We found that HPI axis receptors are required to facilitate light adaptive behavior and maintain baseline activity. Larvae with mutations in a key HPI axis receptor (*mc2r*, *nr3c1*, but not *nr3c2*) did not increase their locomotor activity levels in darkness after a brief (1–2 min) illumination. The mutants required longer durations of illumination (≥ 6 min) to increase locomotor response in the ensuing darkness (Figs. 4 and 5). Our finding is congruent with another *nr3c1* mutant lines in exon 2 (*nr3c1*^*mn61/mn61*^ or *nr3c1*^*mn62/mn62*^) that showed equivalent locomotion to WT fish in a 7.5-min dark–light repeat paradigm (Supplementary Fig. S7)^57^. In addition, perturbation of an HPI axis receptor (*nr3c1* knockout; *nr3c1*^*mn63/mn63*^ or *nr3c1*^*mn65/mn65*^) led to decreased basal locomotor activity under either constant illumination or darkness (Fig. 3). These findings show that the canonical HPI axis contributes to the efficiency of adaptation to changing light conditions, but the photoadaptation can be slowly achieved in HPI axis mutants when light is provided for a long enough duration (≥ 6 min). Thus, the phasic locomotor response can be achieved even without proper HPI axis activity, while maintaining the tonic (basal) locomotion appropriate for the given illumination required *nr3c1* function (Fig. 3). Our findings contrast a previous study that found an increased circadian locomotor activity in *nr3c1* mutants (*gr*^*s357*^) that carried a missense mutation^58^. This discrepancy may be due to the difference in mutant types. While our *nr3c1* mutants in exon 2 or 5 carried a frameshift mutation that was expected to yield a truncated protein, the DNA-binding function was abrogated or changed in the *gr*^*s357*^ variant, possibly leaving the signaling capacity and protein–protein interaction intact^59^. Importantly, elevated levels of cortisol by microinjection^60^ or 24-h bath application^57,61^ led to increased locomotion during the dark, light, or both phases in 4-dpf larvae. Considering the likely hyper-cortisolemic state of *nr3c1* mutant larvae, the level of cortisol only does not seem to explain the locomotor response during illumination changes. Cortisol levels and how HPI axis sensitivity and activity are established during development would need to be taken into consideration to better understand the behavior.

The same pattern of phasic locomotor response was reproduced in much dimmer light only with higher efficiency in HPI axis mutant animals (Fig. 8 and Supplementary Fig. S86)). Both the dim (20.5 µW cm^−2^; ~ 300 lx) and high (469.4 µW cm^−2^; ~ 8000 lx) intensities of white light increased locomotion in darkness post-illumination. Yet, *nr3c1*^*ex5*^ homozygous (HM) mutant larvae achieved WT-level activity after 2 min in the dim illumination, compared to 6–7.5 min they needed in brighter light. Thus, light adaptation was facilitated not only by the number of photons, but also by the appropriate intensity of light. In most animals, the illumination during twilight (dawn, dusk) is a salient zeitgeber (signals that entrain the circadian rhythm)^62,63^. The electroretinogram (ERG) wave patterns change in birds during this period^64^, and there may be distinct photopigments detecting twilight illumination^65^. During the twilight period, animals detect changes in the amount and composition (a shift toward more blue spectrum) of illumination and the angle to the sun. Since the amount (intensity) of illumination is the only variable that changed in this experiment, it is unclear whether the increased efficiency in the light adaptive behavior is related to the twilight-time effect. Further investigation is required for the relationship between light adaptation and illumination intensity. *nr3c1* WT and HM siblings showed a marginal increase in locomotion (< 4 mm/min) after 1-min illumination in dim light (Fig. 8Ab). That was likely to be noise since those low levels of locomotion (effect size) were within the range of basal locomotion, rather than a response to light changes. The likely reason why general WT stock fish showed a more robust response (Supplementary Fig. S86A) than the WT siblings of the *nr3c1* fish (Fig. 8A) after 1-min dim illumination may be familial difference in background genetics. Such familial differences are common among different zebrafish lineages in the laboratory and wild strains^66–70^.

The characterization of the locomotor response in this study reveals complex behavior over time. In the earlier studies using ultrahigh-speed cameras, larvae showed a sharp increase in locomotion immediately after transitioning from dark to light or from light to dark in millisecond scales^53^. These reflexive movements were classified into different categories based on the nature of stimuli, the characteristics of the movement repertoire, and the underlying neural circuits responsible for the movements. The auditory startle, visual startle, dark-flash, and light-flash responses, to name a few, could be differentiated and were comprised of distinct basic units of locomotion^32,71–80^. Our study characterized the rapid non-genomic locomotor responses in the timescale of hormonal response^81–83^ that followed the immediate reflexive responses. The adaptive response to changing illumination arose with or without proper HPI axis receptors activity, demonstrating that vital adaptations essential to the organism would occur by means of multiple redundant pathways. Nevertheless, the canonical HPI axis, as the backbone of the stress response (SR), appeared to play a critical facilitative role (based on the observation in key receptor mutants), making such locomotor adaptation more efficient.

Importantly, a functioning HPI axis receptor (*nr3c1*) was necessary to maintain basal locomotor activity, which implies that dysfunction could be observed in the baseline activity before maladaptive phasic responses. Decreased basal locomotion in *nr3c1*^*ex5*^ HM larvae in light and darkness may be related to the role of GCs as the key signaling molecule in synchronizing the circadian cycle in mammals^9–11,18^. Compared to mammalian systems that rely heavily on the SCN, the circadian regulation is more complex in teleost fish as a range of tissues including the skin, brain, pineal gland, and heart are directly responsive to light and autonomously maintain the circadian clock^84–88^. A broad spectrum of light (ultraviolet, visible, and infrared light) could directly induce clock gene expression in zebrafish cell culture while infrared light could not phase-shift the circadian clock^84^. Despite such complex interaction between the light and clock systems in zebrafish, the genetic abrogation of DNA-binding in *gr*^*s357*^ did not change clock-related gene expression levels while increasing basal locomotor activity^58^. Blocking glucocorticoid (GC) signaling did not change the circadian fluctuation of *crh* (corticotropin releasing hormone) in the neurosecretory preoptic area^30^ (zebrafish homolog to the mammalian hypothalamus)^89^. GC signaling was instead necessary to maintain the level of overall basal *crh* production in teleost fish (6-dpf zebrafish larvae^30^, adult rainbow trout^90^, and adult zebrafish^91^), contrasting the negative feedback of the phasic GC signaling against *crh* production^30^. However, microinjection of anti-cortisol antibody or cortisol to 1-cell stage embryos to deplete or increase cortisol levels led to increased or decreased *crf* expression, respectively, on 4-dpf zebrafish larvae^92^, which demonstrated the opposite trends in basal *crf* levels compared to other studies. It appears that the total amount of cortisol, whether the levels fluctuate, and the developmental stage and history of the animal, all factor in the regulation of basal and phasic HPA/I axis activity. Thus, the role of GC/GR signaling and HPA/I axis dynamics in basal physiology needs further investigation and may add insight into maladaptive HPA/I axis function during the phasic responses.

Mutations in *nr3c2*^*ex2*^ (*nr3c2*^*mn67/mn67*^) did not show any consistent direction of influence in behavior. After 2-min illumination, WT fish appeared to move more than their HM siblings whereas, after 4-min illumination, HM siblings moved more (Fig. 6). After 7.5-min illumination, there was no difference in locomotion between the WT and HM siblings. We found no main effect of homozygosity on locomotion in any of these apparent increases and decreases of locomotion among *nr3c2* fish (main effect of homozygosity, *p* = 0.104, *p* = 0.0728, and *p* = 0.758, in 2-, 4-, and 7.5-min illumination assays, respectively). We previously reported that *nr3c2* knockout does not affect locomotor response after a short (1-min) illumination^23^. Thus, it appears that *nr3c2* does not play a critical role in the photo-adaptive behavior. Our findings contrast a previous study where *nr3c2* mutant larvae showed a significantly increased total locomotion during the dark phase^57^. Such difference may arise due to the husbandry with which *nr3c2* mutants were maintained and the genetic status of the fish (i.e., F2, F3) used in the experiments. While our *nr3c2* lines were maintained as outbred heterozygous fish, adult *nr3c2* homozygous mutants were maintained via inbreeding in the other study. We obtained WT, HT, and HM siblings by crossing heterozygous parent pairs while the other study utilized WT and HM offspring obtained by separately crossing WT and HM adult stock fish. This could also indicate a maternal role for MR, as the resultant larvae studied were maternal-zygotic mutants. Other adult *mr* mutant teleost (medaka; *Oryzias latipes*) showed defects in visuo-locomotor function, failing to closely trace a moving dot^93,94^. In teleosts, *nr3c2* is highly expressed in the brain, evolutionarily more ancient than *nr3c1*, more likely to change expression levels when cortisol levels are altered, involved in the regulation of HPI axis gene expression, and evolutionarily preserved despite evidence for the lack of mineralocorticoid activity^57,91,94,95^. While this line of evidence implies a possible ‘central’ function of Nr3c2 in the brain, what types of behavioral response and what stages of the stress response are regulated by the receptor requires further investigation.

The locomotor response of zebrafish larvae to exogenous stimuli is not normally distributed (non-normal). A large proportion of fish do not move at all or move very little (many zero and negligible values) while a small minority show excessive movement (outliers). The variation in the response is not homogeneous among experimental groups (heteroscedasticity). There is no adequate method to easily transform the response variable to near normal at the individual fish level^96–99^. By comparing the single values averaged over a stretch of time (i.e., total movement during the experimental period), we miss the dynamics of the response over time^100,101^. Such data structures and over-simplification violate key assumptions on which common parametric tests and linear models depend to make valid hypothesis tests^102–104^. Non-normality and violation of homoscedasticity can be addressed, to some degree, by increasing the sample size^99,105^, using the sample means (sampling distribution rather than raw data from individual fish)^105–107^, and including the variation in each experiment as random effects in mixed model approaches^98^. The size of the samples that yield reliably accurate hypothesis tests in non-normal distribution is often less than 100. Even with extremely non-normal and heteroscedastic data sets, a less than 500 sample can perform statistical tests based on linear regression models^105^. However, it is reported that whereas increasing sample size could address non-normality issues more easily, other types of the assumption violation including homoscedasticity, extreme outliers, and independence of errors may persist despite the increased sample size^99^. Moreover, outcome transformations change the result estimates and may bias the result^99^.

To address these issues, several approaches were proposed in the zebrafish research community^96–98,108^. However, with nonparametric tests using rank comparison, we forgo quantitative estimates of the response although the inference may be more justifiable^109^. When a generalized linear model analyzes the proportion of the response vs. nonresponse, we do not show the effect size but just summarize the presence or absence of the response. While nonlinear models effectively characterize the properties of the response (i.e., the rate of increase), the rigidity of the underlying equation limits the applicability of a model to a few specific phenomena. In our case, it was difficult to apply the same nonlinear model to the two distinct response profiles during the dark or light photo period. Thus, it is unlikely that a single solution will be able to address all the assumption violations in the zebrafish locomotor data and produce the right inference. Rather, it would be more important to describe the outcome and its effect size in accordance with the scientific question and discuss the biological relevance of the inference^110–114^. For example, what to report should be determined by the research interest, when a group of zebrafish consistently moved 10 mm per minute making a 100-mm displacement for 10 minutes and another group moved 90 mm for a minute and stayed without movement for 9 minutes. Whether comparing the total distance of 100 mm vs. 90 mm or the pattern of evenness vs. dynamic change is more relevant should be determined by the scientific question in hand. The statistical assumptions violated or satisfied should be chosen by the same standard.

To describe zebrafish locomotor response to changing light, we chose to adopt a statistical model, not commonly used in zebrafish behavioral studies. A generalized additive model (GAM) provided a practical and expandable framework^115,116^, successfully describing locomotor response throughout the entire experimental process. A GAM is an extended generalized linear model (GLM) with a linear predictor that is composed of a sum of smooth functions of the explanatory variable (smooth terms) in addition to the parametric explanatory variables^43,45,117^. A range of link functions can be used to linearly relate the predictors to the response variable of an exponential or some non-exponential family distribution. However, we focused on estimating the effect size and did not use additional link function (used the identity link function). The variance among the individual assays was modeled as a random effect. The simplicity and flexibility of the GAMs allow describing nonlinear relationships in the linear additive framework. In our GAM analysis pipeline, the main effect of the explanatory variable for the whole experiment was first evaluated, followed by post hoc pairwise analyses at each second that reported the significance of the difference of the means between the compared pair. Then, the proportion of significant difference between each pair was compared among different assay regimens to see if different durations of illumination led to distinct locomotor response compared to another. The analysis pipeline allowed quantitative estimation of the effect of the explanatory variable at each second without losing the dynamics of changes in locomotion and the effect of different assay regimen on locomotion.

The body’s homeostasis represents a point in the continuum of physiological and behavioral states in an organism. Allostasis describes how a homeostatic point is determined in response to the changing environment. The redundancy in the SR ensures an adequate adaptation even if a defective pathway is present. However, the compensatory pathways in achieving allostasis may be biologically expensive over time, eventually leading to maladaptation. In our investigation, the canonical HPI axis receptors were dispensable to mounting light adaptive phasic responses when the illumination duration was long enough and the intensity was optimal. However, maintaining light adaptive tonic states at the baseline required *nr3c1* function. Our findings imply that both phasic and tonic responses of the HPA/I axis need to be investigated to understand the stress response. Stress-aggravated psychiatric and metabolic disorders may arise not only from the aftereffect of HPA/I axis activity (hyper- or hypo-cortisolemia) but also from the peripheral roles of glucocorticoid signaling in relevant organ systems where cortisol and its cognate receptors support a wide variety of adaptive responses.

### Supplementary Information

Supplementary Information.

## Data Availability

All primary data and supplementary materials were deposited in the open access data repository ( Enter: "10.6084/m9.figshare.25467865" at figshare.com). The core R scripts for GAM modeling were deposited to GitHub.com (https://github.com/moonlarkalto/HPA_gam) and the data sets for test-running the R scripts were deposited to FigShare.com in the same link.

## Abbreviations

ACTH: Adrenocorticotropic hormone
CR: Corticosteroid receptor; includes GR and MR
CRH: Corticotropin releasing hormone
GAM: Generalized additive model
GC: Glucocorticoids; cortisol, corticosterone
GR: Glucocorticoid receptor
HM: Homozygous/homozygotes
HPA: Hypothalamic–pituitary–adrenal
HT: Heterozygous/heterozygotes
HPI: Hypothalamic–pituitary–interrenal
IR: Infrared
KO: Knockout
mc2r: Melanocortin receptor type 2; ACTH receptor
MR: Mineralocorticoid receptor
nr3c1: Nuclear receptor subfamily 3 group c member 1; glucocorticoid receptor, GR
nr3c2: Nuclear receptor subfamily 3 group c member 2; mineralocorticoid receptor, MR
SR: Stress response
TALEN: Transcription activator-like effector nuclease
WT: Wild type
Zeitgeber: Environmental signals that entrain the circadian rhythm in an organism
