# Supplementary material for: Key HPI axis receptors facilitate light adaptive behavior in larval zebrafish
Source: Sci Rep. 2024 Apr 2;14:7759. doi: 10.1038/s41598-024-57707-6 (PMC10987622; doi:10.1038/s41598-024-57707-6)
Supplement: Supplementary file 1 — Supplementary Information. [file 41598_2024_57707_MOESM1_ESM.zip › Supp_Figs_SciRpts/SuppFigS76_dim_WT_4x[7.5_2min]_gamCheck.pdf]

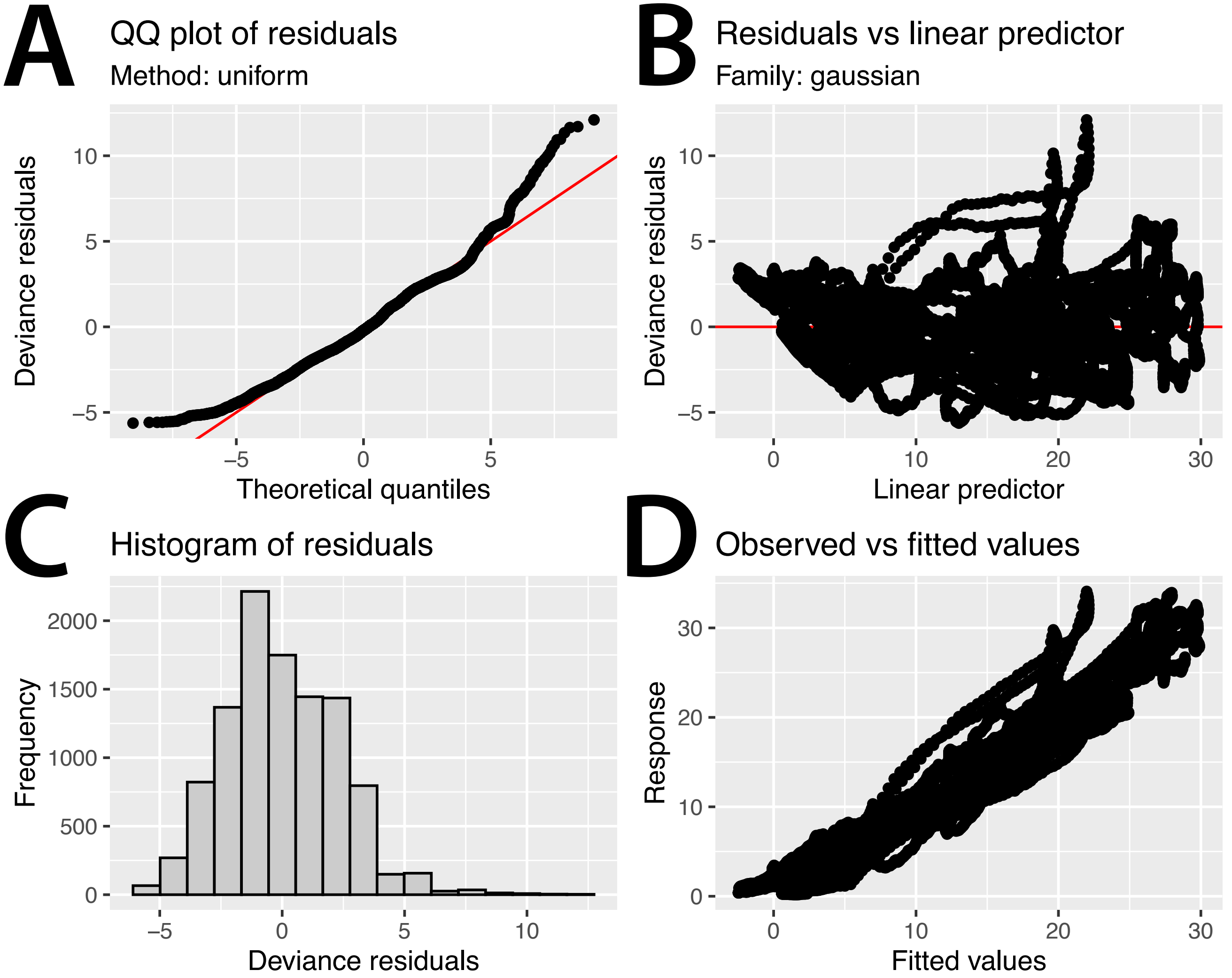

Supplementary Figure S76. Fitness of the generalized additive model for locomotor response of WT larvae in 4x[7.5 dark + 2-min light] assay. A QQplot of residuals. **B** Residuals vs. linear predictor. **C** Histogram of residuals. **D** Observed vs. fitted value.
