# Supplementary material for: Key HPI axis receptors facilitate light adaptive behavior in larval zebrafish
Source: Sci Rep. 2024 Apr 2;14:7759. doi: 10.1038/s41598-024-57707-6 (PMC10987622; doi:10.1038/s41598-024-57707-6)
Supplement: Supplementary file 1 — Supplementary Information. [file 41598_2024_57707_MOESM1_ESM.zip › Supp_Figs_SciRpts/SuppFigS86_dim_WT.pdf]

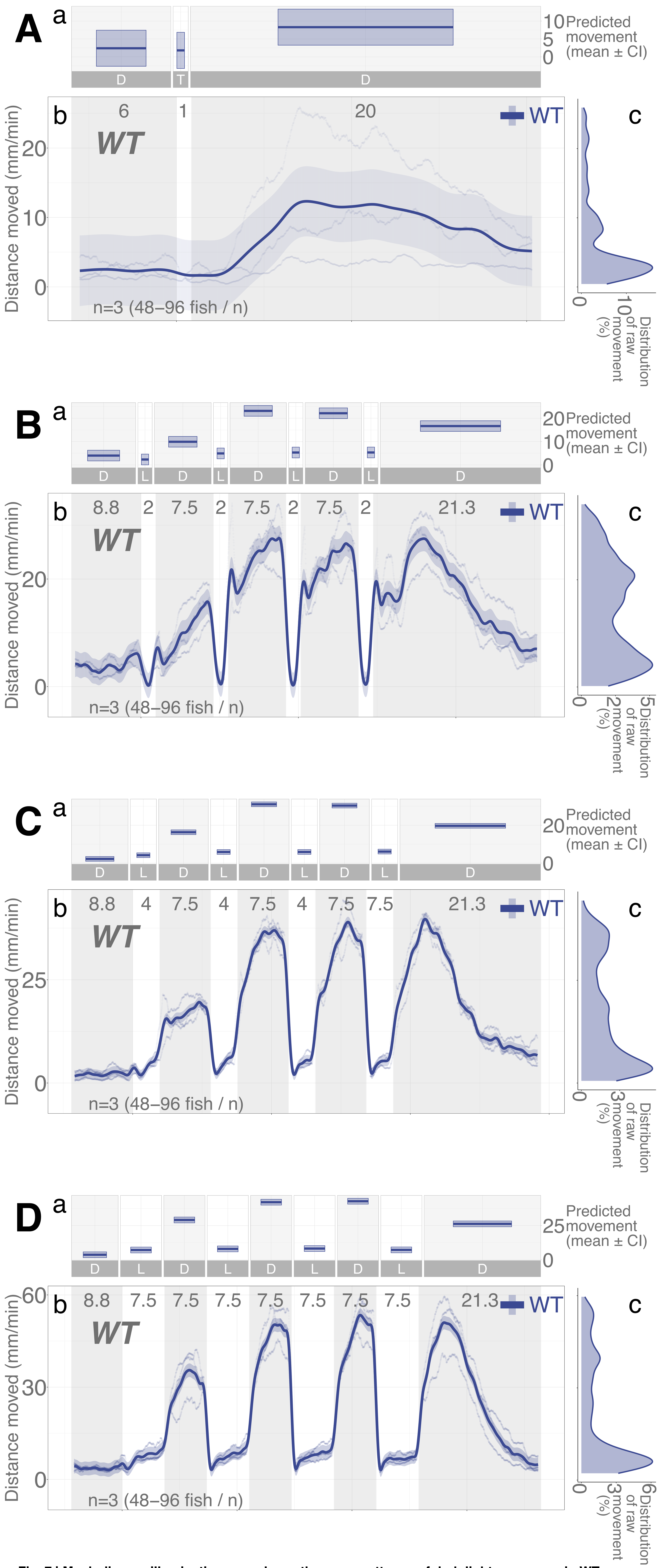

Fig. 7 | Much dimmer illumination reproduces the same patterrrn of dark-light responses in WT larvae. Dimmer illumination (20.5 μW•cm⁻²; 300 lx) was used compared to that of all other experiments (469.4 μW•cm⁻²; 8000 lx). IR illumination was the same (116.0 μW•cm⁻²; 0 lx). **Aa, Ba, Ca, Da** Locomotor activity (mean predicted value [mm/min] ± 95%CI) predicted by the GAM for each photo period (gray: dark, whie: light period). A brief illumination assay (1-min light) without the repeat components was included to understand behavior in dim light (A). **Ab, Bb, Cb, Db** Locomotor response of WT larvae during dark-light repeat assays. The scatterplot (points) shows actual mean locomotor activity (mm/min) of each assay. The line graph shows predicted locomotor activity by the GAM (predicted value ± 95%CI; gray: dark, white: light period). **Ac, Bc, Cc, Dc** Density disbution of actual mean locomotor activity shows right skewed distribution, but the degree of skewness is less than that in other assays. The integration of the curve equals 100 %. (D: dark, L: light)
