# Supplementary material for: Key HPI axis receptors facilitate light adaptive behavior in larval zebrafish
Source: Sci Rep. 2024 Apr 2;14:7759. doi: 10.1038/s41598-024-57707-6 (PMC10987622; doi:10.1038/s41598-024-57707-6)
Supplement: Supplementary file 1 — Supplementary Information. [file 41598_2024_57707_MOESM1_ESM.zip › Supp_Figs_SciRpts/SuppFigS88_baseline_nr3c1.pdf]

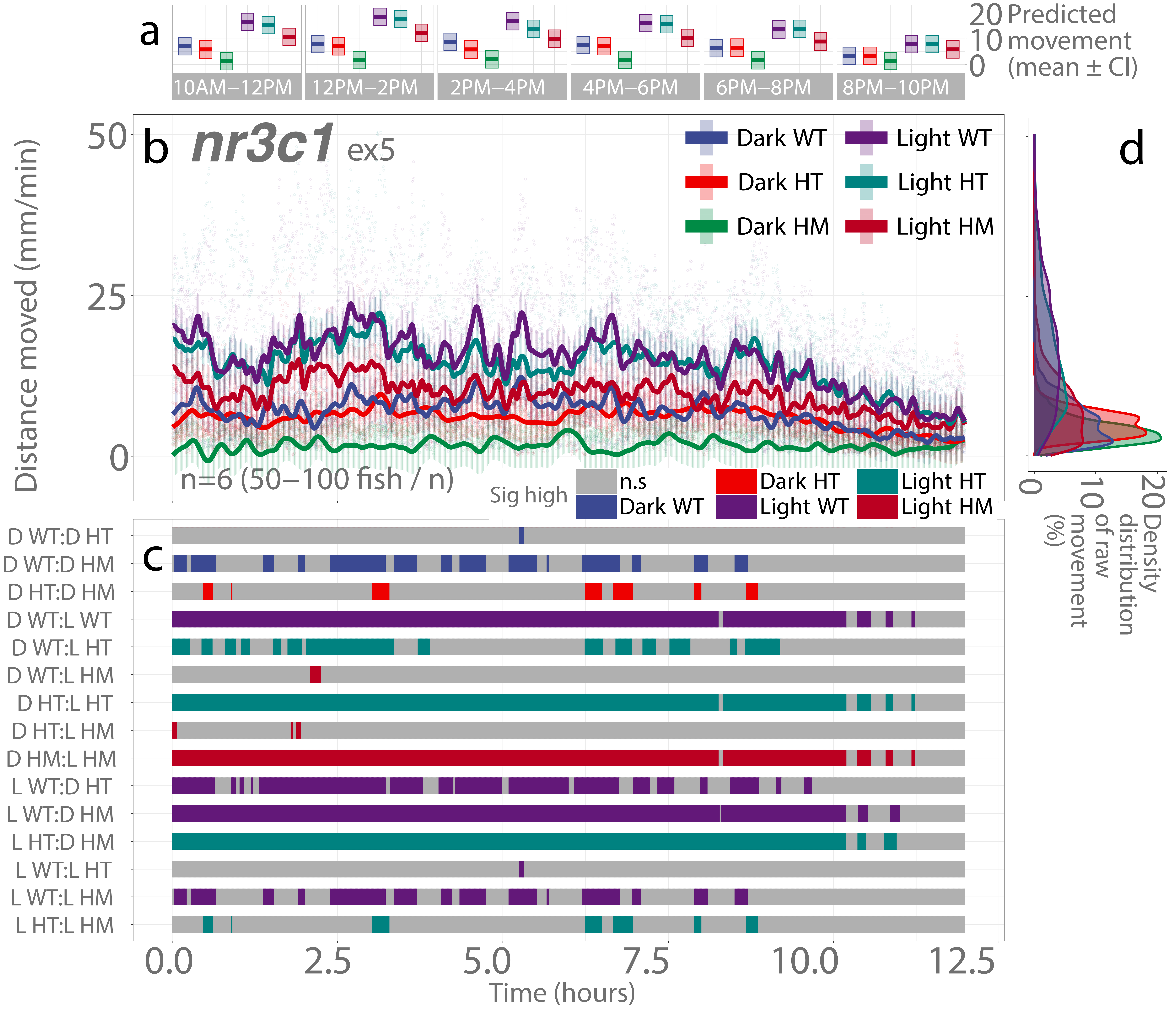

Supplementary Fig. 88 | Illumination and genotype are determinants of basal locomotion in *nr3c1* knockout lineage larvae. a Locomotor activity (mean predicted value (mm/min) ± 95%CI) for each experimental condition predicted by the GAM for each time span. **b** Basal locomotor activity of nr3c1ex5 larvae over 12 hours. The scatterplot (points) shows actual mean locomotor activity (mm/min) for each experimental condition of each assay. The line graph shows predicted locomotor activity for each experimental condition by the GAM (predicted value ± 95%CI). **c** Time points where an experimental condition showed significantly high locomotor activity compared to the other in a pairwise comparison. The color shown indicates the group with a significantly higher outcome in a pairwise comparison. **d** Histogram of individual fish locomotion (y axis is the same as subpanel b). Density distribution of actual mean locomotor activity shows severely right skewed distribution (low locomotor response). The integration of the curve equals 100%. (D: dark, L: light, WT: wildtype, HT: heterozygous, HM: homozygous, n.s: not significant, *nr3c1* genotypes: $nr3c1^{+/+}$ [WT], $nr3c1^{+/mn63}$ [HT], $nr3c1^{mn63/mn63}$ [HM, KO], or $nr3c1^{+/mn65}$ [HT], $nr3c1^{mn65/mn65}$ [HM, KO])
