# Supplementary material for: Key HPI axis receptors facilitate light adaptive behavior in larval zebrafish
Source: Sci Rep. 2024 Apr 2;14:7759. doi: 10.1038/s41598-024-57707-6 (PMC10987622; doi:10.1038/s41598-024-57707-6)
Supplement: Supplementary file 1 — Supplementary Information. [file 41598_2024_57707_MOESM1_ESM.zip › Supp_Figs_SciRpts/SuppFigS05_IR_Med.pdf]

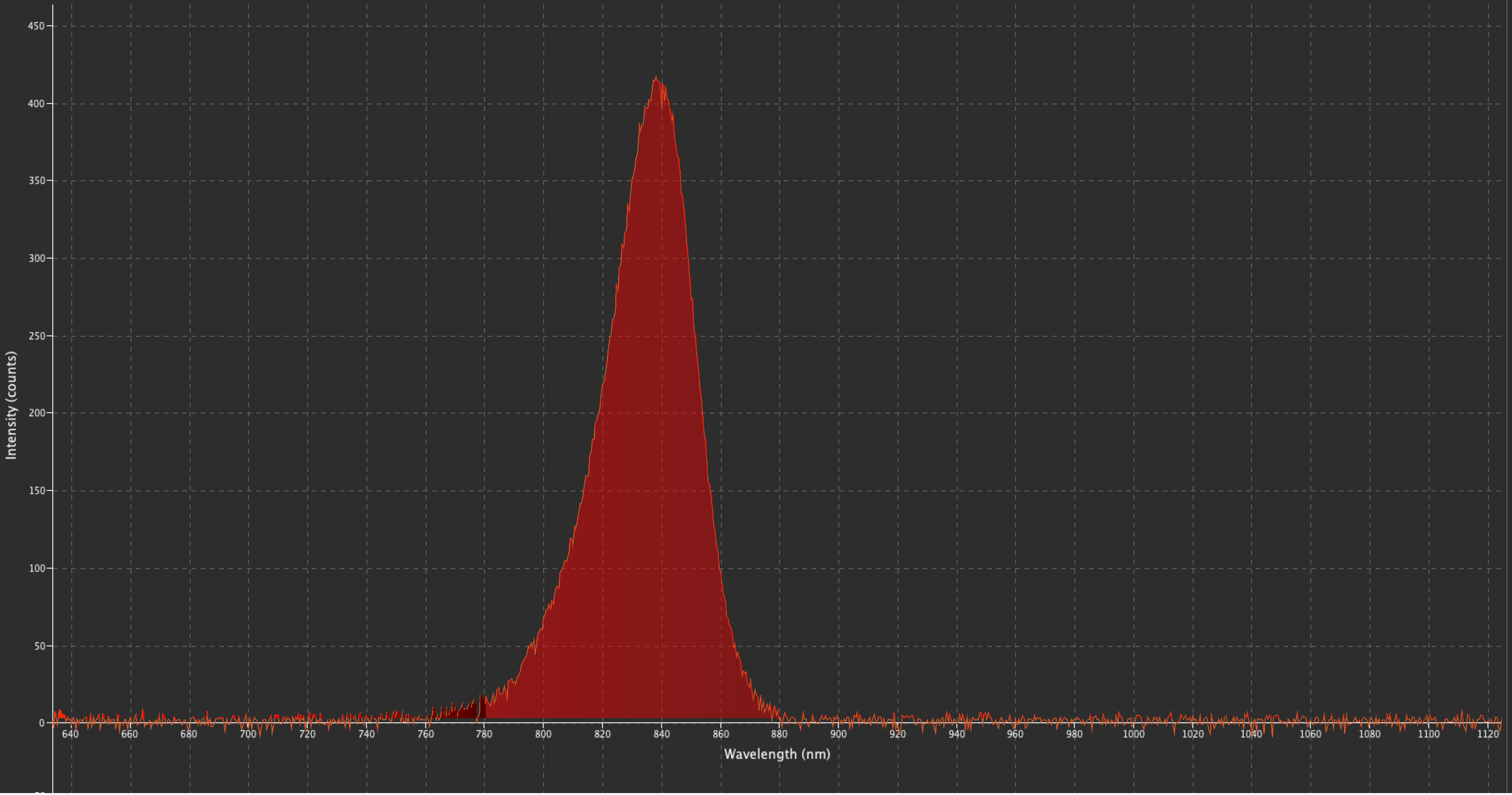

Supplementary Figure S5. Light spectrum and relative intensity of infrared light at the medium intensity setting. Light spectrum and relative intensity were measured by a spectrometer (STS-NIR, Ocean Optics Inc.). The graph was pseudocolored for the optimal detection range of the spectrometer with the spectrometer printing option.
