# Supplementary material for: Key HPI axis receptors facilitate light adaptive behavior in larval zebrafish
Source: Sci Rep. 2024 Apr 2;14:7759. doi: 10.1038/s41598-024-57707-6 (PMC10987622; doi:10.1038/s41598-024-57707-6)
Supplement: Supplementary file 1 — Supplementary Information. [file 41598_2024_57707_MOESM1_ESM.zip › Supp_Figs_SciRpts/SuppFigS43_dim_nr3c1.e5_4x[7.5_2min]_prop_each.pdf]

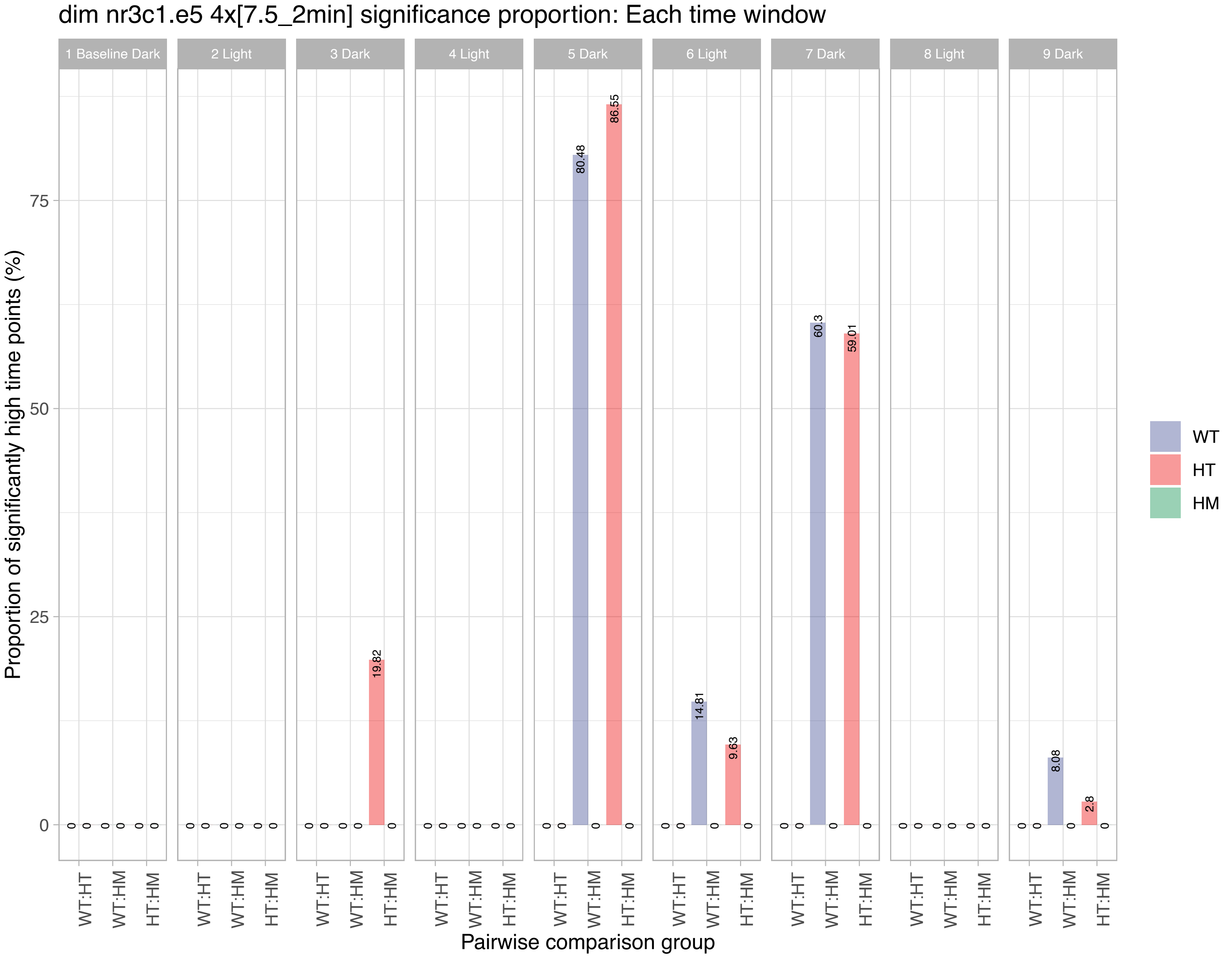

Supplementary Figure S43. Summary proportions (%) of locomotor response of *nr3c1*^{ex5} lineage fish for each photo period of 4x[7.5 dark + 2-min dim light] assay. The proportion of time where the larvae of a condition moved significantly more than those in the other condition in pairwise comparison. Proportion is computed for each photo period. (WT: wildtype, HT: heterozygous, HM: homozygous)
