# Supplementary figures and images for: Key HPI axis receptors facilitate light adaptive behavior in larval zebrafish
Source: Sci Rep. 2024 Apr 2;14:7759. doi: 10.1038/s41598-024-57707-6 (PMC10987622; doi:10.1038/s41598-024-57707-6)

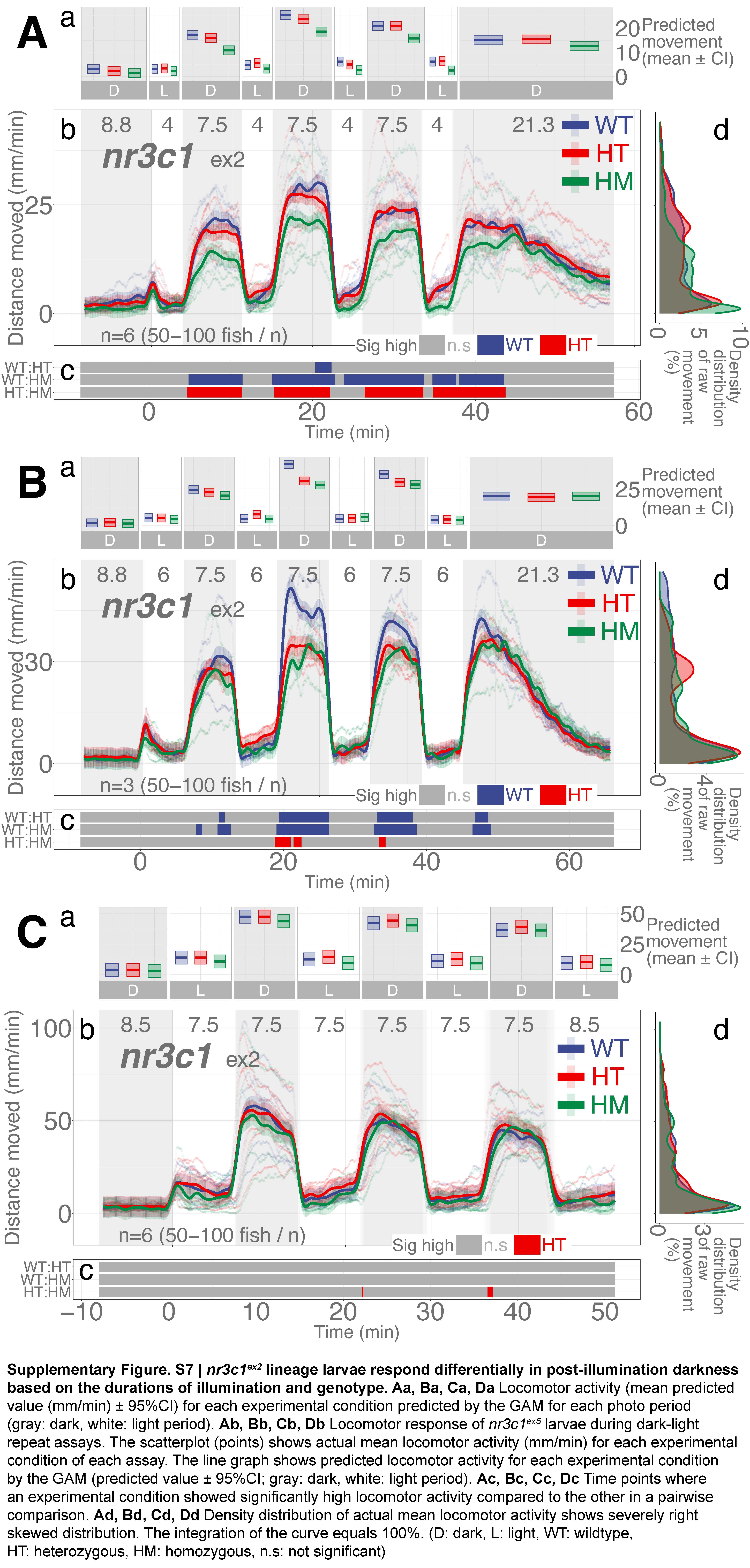

Supplement: Supplementary file 1 — Supplementary Information. [file 41598_2024_57707_MOESM1_ESM.zip › Supp_Figs_SciRpts/SuppFigS07_nr3c1.e2_response.jpg]
