# Supplementary material for: Key HPI axis receptors facilitate light adaptive behavior in larval zebrafish
Source: Sci Rep. 2024 Apr 2;14:7759. doi: 10.1038/s41598-024-57707-6 (PMC10987622; doi:10.1038/s41598-024-57707-6)
Supplement: Supplementary file 1 — Supplementary Information. [file 41598_2024_57707_MOESM1_ESM.zip › Supp_Figs_SciRpts/SuppFigS87_gr.mrKO_1min_response.pdf]

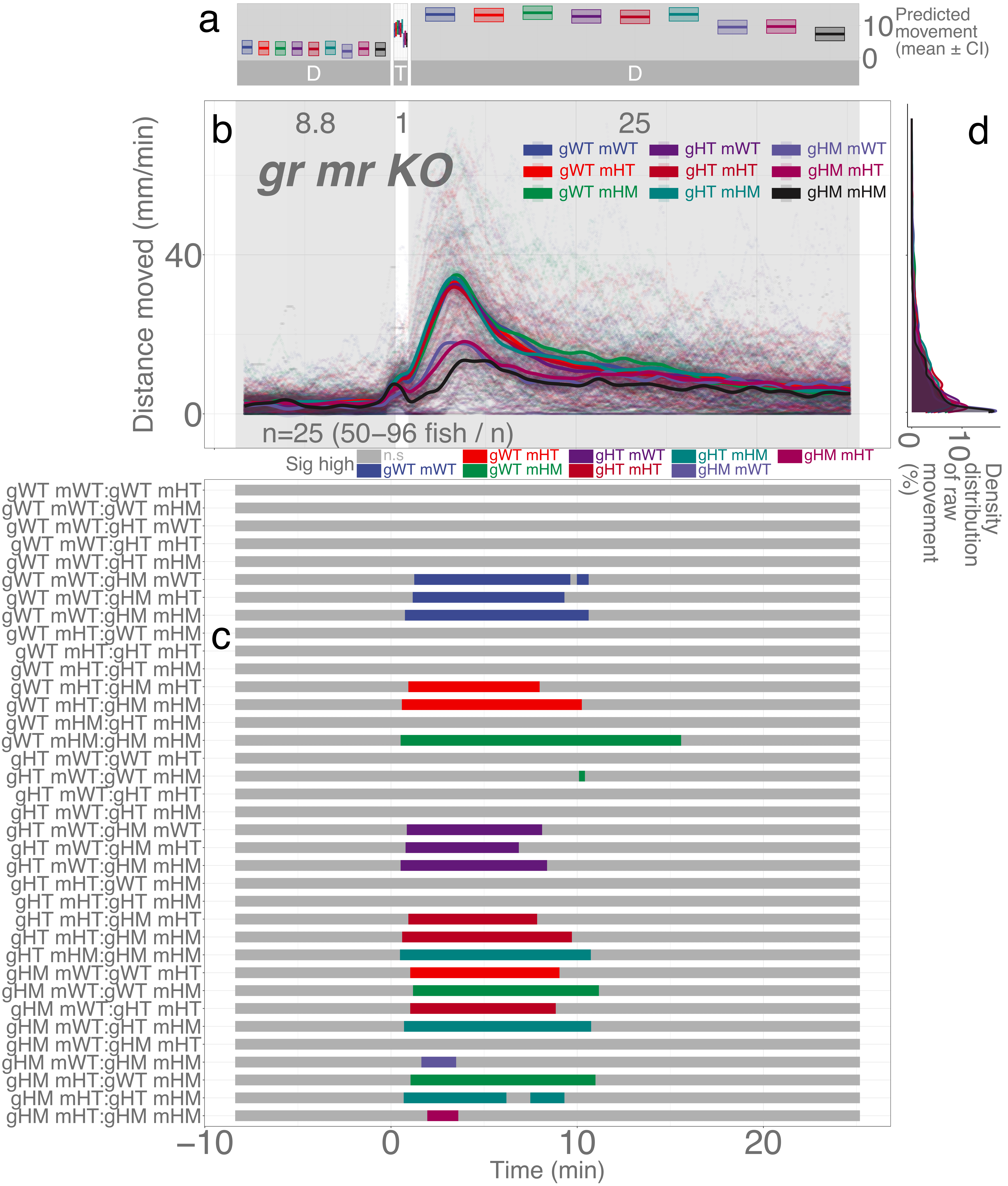

Supplementary Figure. S87 | *nr3c1* (gr) and *nr3c2* (mr) double knockout lineage larvae show varying locomotor activity levels in the darkness after short illumination (1-min) a Locomotor activity (mean predicted value) (mm/min) ± 95%CI) for each genotype (gr WT mr WT, gr WT mr HT, gr WT mr HM, gr HT mr WT, gr HT mr HT, gr HT mr HM, gr HM mr WT, gr HM mr HT, gr HM mr HM), predicted by the GAM. The full genotypic combinations are shown in this figure while only WT and HM are presented in Supplementary Fig. S86 to increase readibility. The underlying data set is the same in the two figures. **b** Locomotor response in the baseline (dark) and post-illumination (dark). The scatterplot (points) shows actual mean locomotor activity (mm/min) for each experimental condition of each assay. The line graph shows predicted locomotor activity for each genotype by the GAM (predicted value ± 95%CI). **c** Time points where a genotype showed significantly high locomotor activity compared to the other in a pairwise comparison. **d** Density distribution of actual mean locomotor activity shows severely right skewed distribution. The integration of the curve eaquals 100% (gr: glucocorticoid receptor (*nr3c1*), mr: mineralocorticoid receptor (*nr3c2*), D: dark, T: treatment [illumination], WT: wildtype, HT: heterozygous, HM: homozygous, n.s: not significant, *nr3c1* genotypes: $nr3c1^{+/+}$ [WT], $nr3c1^{+/mn63}$ [HT], $nr3c1^{mn63/mn63}$ [HM, KO], or $nr3c1^{+/mn65}$ [HT], $nr3c1^{mn65/mn65}$ [HM, KO], *nr3c2* genotypes: $nr3c2^{+/+}$ [WT], $nr3c2^{+/mn67}$ [HT], $nr3c2^{mn67/mn67}$ [HM, KO])
